# Burkitt lymphoma with a granulomatous reaction: an M1/Th1‐polarised microenvironment is associated with controlled growth and spontaneous regression

**DOI:** 10.1111/his.14391

**Published:** 2021-07-05

**Authors:** Massimo Granai, Stefano Lazzi, Virginia Mancini, Ayse Akarca, Raffaella Santi, Federica Vergoni, Ester Sorrentino, Raffaella Guazzo, Lucia Mundo, Gabriele Cevenini, Claudio Tripodo, Gioia Di Stefano, Benedetta Puccini, Maurilio Ponzoni, Elena Sabattini, Claudio Agostinelli, Nuray Bassüllü, Tülay Tecimer, Ahu Senem Demiroz, Leah Mnango, Stephan Dirnhofer, Leticia Quintanilla‐Martinez, Teresa Marafioti, Falko Fend, Lorenzo Leoncini

**Affiliations:** ^1^ Department of Medical Biotechnologies University of Siena Siena Italy; ^2^ Institute of Pathology University of Tübingen Tübingen Germany; ^3^ Department of Cellular Pathology University College London London UK; ^4^ Department of Pathology University of Florence Florence Italy; ^5^ Health Research Institute University of Limerick Limerick Ireland; ^6^ Department of Human Pathology University of Palermo Palermo Italy; ^7^ Department of Haematology University of Florence Florence Italy; ^8^ Department of Pathology University Vita‐Salute San Raffaele Milano Italy; ^9^ Haemolymphopathology Unit ‐ IRCCS ‐ Azienda Ospedaliero‐Universitaria di Bologna Bologna Italy; ^10^ Department of Pathology Bilim University İstanbul Turkey; ^11^ Department of Pathology Acibadem University İstanbul Turkey; ^12^ Department of Pathology İstanbul University Cerrahpaşa İstanbul Turkey; ^13^ Department of Pathology Muhimbili National Hospital and University for Healthcare and Allied Sciences Dar‐es‐Salaam Tanzania; ^14^ Institute of Pathology University Hospital Basel Basel Switzerland

**Keywords:** Burkitt lymphoma, EBV, granulomatous reaction, *in‐situ* lymphoid neoplasia, microenvironment, M1 polarised macrophages, Th1 T cells

## Abstract

**Aims:**

Burkitt lymphoma (BL) is an aggressive B‐cell lymphoma that, in some instances, may show a granulomatous reaction associated with a favourable prognosis and occasional spontaneous regression. In the present study, we aimed to define the tumour microenvironment (TME) in four such cases, two of which regressed spontaneously.

**Methods and results:**

All cases showed aggregates of tumour cells with the typical morphology, molecular cytogenetics and immunophenotype of BL surrounded by a florid epithelioid granulomatous reaction. All four cases were Epstein–Barr virus (EBV)‐positive with type I latency. Investigation of the TME showed similar features in all four cases. The analysis revealed a proinflammatory response triggered by Th1 lymphocytes and M1 polarised macrophages encircling the neoplastic cells with a peculiar topographic distribution.

**Conclusions:**

Our data provide an *in‐vivo* picture of the role that specific immune cell subsets might play during the early phase of BL, which may be capable of maintaining the tumour in a self‐limited state or inducing its regression. These novel results may provide insights into new potential therapeutic avenues in EBV‐positive BL patients in the era of cellular immunotherapy.

## Graphical abstract

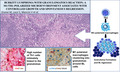

## Introduction

Burkitt lymphoma (BL) is an aggressive B‐cell lymphoma characterised by diffuse proliferation of medium‐sized monomorphic lymphoid cells with basophilic cytoplasm and cohesive growth. A so‐called starry sky pattern is usually present, owing to the presence of numerous macrophages with tangible apoptotic bodies. However, some cases show a florid granulomatous reaction and are characterised by a favourable outcome and, in rare instances, spontaneous regression.^1^, ^2^, ^3^, ^4^, ^5^, ^6^ More recently, this issue was discussed in the Lymphoma Workshop (LYWS) of the 18th meeting of the European Association for Haematopathology (EAHP) in 2016 in Basel. BL cases with a granulomatous reaction were presented, two of which showed spontaneous remission without further therapy after needle core biopsy or lymph node excision.^7^ The almost identical morphological features of these cases may provide a link between the excellent prognosis of these patients and the peculiar immune reaction of the host, highlighting the need for further investigations with comprehensive characterisation of the tumour microenvironment (TME). In fact, none of the previous studies has characterised the nature of the inflammatory infiltrate and its polarisation towards an activated (proinflammatory) or tolerant (protumoral) state.

Only in the last decade has the cellular background in which lymphoma cells thrive become an important target of inquiry. The functions of what used to be considered passive bystanders are quickly becoming elucidated, in order to provide potential targets for immunotherapy. In recent years, a model has been developed to describe the complex mechanism of macrophage activation as polarisation towards two opposite states, namely M1 and M2, with proinflammatory and protumoral properties respectively. The M1/M2 nomenclature has been inspired by the Th1 versus Th2 concept.^8^

Th1 lymphocytes and M1 macrophages are the primary sources of proinflammatory cytokines, which also promote cancer immunosurveillance and cytotoxicity. On the other hand, these effects are counterbalanced by M2 macrophages, Th2 lymphocytes and regulatory T cells (Tregs) having anti‐inflammatory and protumoral effects.^9^ In particular, Th1 cells drive the type 1 pathway (‘cellular immunity’) to fight viruses and eliminate cancerous cells, whereas Th2 cells drive the type 2 pathway (‘humoral immunity’), up‐regulating antibody production.^9^

Differentiation of CD4+ T cells into Th1 and Th2 effector cells is controlled by the transcription factors T‐box protein expressed in T cells (T‐bet; also called TBX21) and GATA‐binding protein 3 (GATA3), respectively. T‐bet overexpression causes differentiation into the Th1 lineage, whereas loss of T‐bet expression induces default commitment to the Th2 and Th17 lineages.^10^ T‐bet directly activates interferon (IFN)‐γ gene transcription, and enhances the development of Th1 cells. Therefore, CD4+/T‐bet+ cells are considered to be Th1 cells, whereas CD4+/GATA3+ cells are considered to be Th2 cells.

Th1 lymphocytes are well‐known IFN‐γ secretors, being involved in the regulation of macrophage polarisation, promoting and maintaining the formation of granulomas.^9^, ^11^ IFN‐γ activates the IFN regulatory factor–signal transducer and activator (STAT) (via STAT1) pathway, switching macrophage function towards the M1 phenotype.^11^

However, the immunophenotypic features that distinguish M1 and M2 macrophages are still not standardised.^12^, ^13^ Given the fact that, in recent studies,^13^ the scavenger receptor CD163 alone has not been considered to be a reliable marker of M2 polarisation, the combination of c‐Maf, an essential transcription factor for interleukin‐10 gene expression in macrophages, and CD68 as a generic macrophage marker may be used to identify M2 macrophages. On the other hand, an antibody against phosphorylated STAT1 (pSTAT1) may be used to identify M1 macrophages and IFN‐γ‐primed dendritic cells.

Epstein–Barr virus (EBV)‐specific T‐cell responses in BL have not been fully elucidated. Indeed, Epstein–Barr nuclear antigen (EBNA) 1, which is usually the only latent protein expressed in EBV+ BL, has the ability to inhibit proteasomal processing through its Gly‐Ala repeat domain.^14^ In addition, studies have shown defects in major histocompatibility complex class I antigen processing in BL.^15^ These features are well‐known aspects of BL cells, potentially explaining their escape from immune surveillance.^16^ On the other hand, other authors have shown that EBNA1‐specific CD4+ Th1 cells can be isolated from EBV‐seropositive healthy adults,^17^ and that these cells are cytolytic for BL cell lines constitutively expressing human leukocyte antigen (HLA) class II.^18^, ^19^, ^20^, ^21^

Therefore, the aim of this study was to investigate the immune landscape in four cases of EBV+ BL with a granulomatous reaction, focusing on the quantitative and topographic distribution of the innate immunity [M1/M2 macrophages, plasmacytoid dendritic cells (pDCs), and natural killer (NK) cells] and adaptive immunity (Th1/Th2 lymphocytes and Tregs) cell subsets.

## Materials and methods

A formalin‐fixed paraffin‐embedded (FFPE) BL case was retrieved from the Department of Medical Biotechnologies, University of Siena, Siena, Italy (case 1), and two cases (case 2 and case 3) were retrieved from the LYWS of the 18th EAHP meeting, in Basel, Switzerland, 2016, and were included in the workshop report.^7^ The third case submitted to the LYWS was not included in this study because of lack of available sections. The fourth case (case 4) was retrieved from the 1st Workshop to support the NIHR‐RIGHT Aggressive Infection Related‐East African Lymphoma (AI‐REAL) Study in Dar‐es‐Salaam (Tanzania), in February 2020.

The diagnosis of BL was issued by expert haematopathologists following the criteria described in the 2017 World Health Organization classification of tumours of haematopoietic and lymphoid tissue.^6^ Diagnostic immunohistochemistry was performed on the Bond III Autostainer (Leica Microsystems, Newcastle upon Tyne, UK), according to the manufacturer’s instructions. *In‐situ* hybridisation for EBV‐encoded small RNAs (EBERs) was performed on deparaffinised 4‐mm‐thick FFPE tissue sections with a Dako Detection Kit for EBER (DakoCytomation, Glostrup, Denmark). The slides were counterstained with haematoxylin and fixed in Faramount (DakoCytomation).

Fluorescence *in‐situ* hybridisation for *MYC* rearrangement was performed for each case, with a break‐apart probe (BAP) (Vysis *MYC* Dual Colour Break Apart Rearrangement Probe; Abbott, Wiesbaden, Germany) and a dual fusion probe (ZytoVision GmbH, Bremerhaven, Germany), according to the manufacturer’s instructions. For each specimen, a 4‐µm‐thick tissue section embedded in paraffin was cut. Briefly, the slides were incubated in heat pretreatment solution, washed, digested, dehydrated, and hybridised with probes. At least 100 intact non‐overlapping nuclei were analysed manually on a Leica DM 600B (Leica Microsystems, Heerbrugg Switzerland) fluorescence microscope equipped with 4′,6‐diamidino‐2‐phenylindole, Spectrum Green and Spectrum Orange filters.

The antibody panels used for multiplex immunohistochemistry (mIHC) consisted of eight different triple and double stains: (i) CD68, CD163, and c‐Maf; (ii) programmed death‐ligand 1 (PD‐L1), CD163, and c‐Maf; (iii) CD4, CD25, and forkhead box P3 (FOXP3); (iv) CD8, programmed cell death protein 1 (PD‐1), and lymphocyte‐activation gene 3 (LAG3); (v) pSTAT1, CD68, and CD123; (vi) CD4 and T‐bet; (vii) CD4 and GATA3; and (viii) CD20 and T‐bet (see Table S1 for antibody characteristics). CD56 and CD57 were included as single stains. Double staining and triple staining were performed with an automated staining system (DISCOVERY ULTRA IHC/ISH research platform; Roche Diagnostics, Indianapolis, USA) for open procedures, according to the manufacturer’s protocols.^22^ The detailed protocols for the application of each stain are shown in Doc. S1. The CD68+/pSTAT1+, CD123+/pSTAT1+, CD163+/CD68+/c‐Maf+, CD163+/c‐Maf+, CD4+/T‐bet+, CD4+/GATA3+, CD4+/CD25+/FOXP3+ and CD8+/PD‐1+/LAG3+ cells were evaluated manually and independently by three experienced pathologists (M.G., V.M, and T.M.) by counting the individual cell types in 10 high‐power fields under a × 40 objective 172 (Nikon Eclipse E400, Nikon Corporation, Tokyo, Japan), and taking into account either nuclear and surface stains, reported as percentages for each cell type in scores rounded up to the nearest 5%.^23^, ^24^ Only CD163+/CD68+/c‐Maf+ and CD163+/CD68–/c‐Maf+ cells were considered to be M2 macrophages; CD163–/CD68+/c‐Maf– and CD68+/pSTAT1+ cells were considered to be M1 macrophages, and CD123+/pSTAT1+ cells were considered to be IFN‐γ‐primed pDCs.^25^ The interobserver reproducibility of each cell count was assessed according to the coefficient of variation as a percentage (CV%), which is the ratio of the standard deviation to the mean of the three percentage observations. For each variable, percentage agreement across the four cases analysed was then expressed as the mean of 100 – CV% and its 95% confidence interval, estimated with the bias‐corrected and accelerated bootstrap technique. The percentages of each cell type for each observer and the level of interobserver reproducibility for each variable are shown in Table S2.

Tissue sections from the same set of cases and without antibody/chromogens were used as negative controls. Three tonsils, three reactive lymph nodes and three cases of conventional BL with the typical starry sky pattern were used as positive controls.

## Results

### CLINICAL HISTORY

Case 1 was a 65‐year‐old female with an isolated right axillary lymphadenopathy and no signs of systemic infection. She underwent excision of the enlarged lymph node, and a diagnosis of focal involvement by EBV‐positive B cells with features of BL was made, associated with reactive lymphadenitis, and a granulomatous reaction. The computed tomography–positron emission tomography (CT‐PET) scan showed no evidence of other hypermetabolic lymph nodes. Bone marrow biopsy showed normal haematopoiesis, and flow cytometry analysis showed a normal phenotype. Therefore, the patient was followed closely, and a ‘watch and wait’ strategy was used, without any chemotherapy. The patient was well and in good condition 30 months after the initial diagnosis.

Case 2 was a 16‐year‐old male who presented with recurrent swelling on the left side of the neck. First, he was treated with antibiotic therapy for an upper respiratory tract infection. After 7 months, a diagnosis of viral infection was made, without specific treatment. Because of persistent lymph node enlargement, an excisional lymph node biopsy was performed. The lymph node was 60 mm in diameter, and a diagnosis of BL with a granulomatous reaction was made. The patient had no signs of other lymphadenopathies, splenomegaly, or hepatomegaly. The levels of lactate dehydrogenase (LDH) and β2‐microglobulin were normal. A CT‐PET scan showed no evidence of disease, and the bone marrow biopsy was also negative. The patient refused multi‐agent chemotherapy. After 4 years, he was in complete remission without any treatment.

Case 3 was a 47‐year‐old female who presented with cervical swelling for 1 month without B symptoms. A computed tomography (CT) scan showed a 33 × 24‐mm lymphadenopathy next to the left parotid. Peripheral blood and biochemical parameters were normal, except for LDH: 512 U/l (range, 225–450 U/l). The left submandibular lymph node was excised for further investigation, and a diagnosis of BL with granulomatous reaction was proposed. Multi‐agent chemotherapy was initiated. After three cycles of chemotherapy, the patient was in complete remission, and after 2 years of follow‐up he showed no evidence of disease.

Case 4 was a 12‐year‐old male from Kigoma, Tanzania. He was admitted to the local hospital for a swelling in the right lower jaw region that had persisted for >2 months. Prior to the swelling, there was a history of trauma followed by mobility of the lower jaw and regional pain. After tooth extraction, there was no reduction in the symptoms and there was a further increase in the swelling. The patient was sent to a reference centre for further management. The CT scan of the head showed features suggestive of BL in the right lower jaw region, mild right maxillary sinusitis, no evidence of cervical or mediastinal lymphadenopathy, and no evidence of brain involvement. Surgical enucleation and curettage of the tumour was performed. After six cycles of chemotherapy with rituximab, cyclophosphamide, oncovin (vincristine), doxorubicin, and methotrexate, the patient showed no residual disease and was discharged. After 16 months of follow‐up, the patient was asymptomatic.

### MICROSCOPIC AND IMMUNOHISTOCHEMICAL FINDINGS

The morphological and immunophenotypic features of all four cases are summarised in Table 1. The common features of all the cases in the study were the presence of focal or diffuse infiltration by BL cells, and a prominent granulomatous reaction. All cases were EBV‐positive with latency I.

**Table 1:** Clinical and immunophenotypic features

Case	Age (years)/sex	Location	Stage	Treatment	Prognosis	EBV	MYC protein expression	*MYC* translocation
Case 1	65/F	Right axillary lymph node	I	None	Healthy after 30 months of follow‐up	IHC: EBNA1+ (LMP1–, LMP2–, EBNA2–, BZLF1–)	Yes (100%)	t(8;14) break‐apart and fusion probes
Case 2: LYWS‐353	26/M	Mandibular lymph node	I	None	Healthy after 48 months of follow‐up	IHC: EBNA1+/BZLF1–	Yes (100%)	t(8;14) break‐apart and fusion probes
Case 3: LYWS‐360	47/F	Cervical lymph node	I	Three cycles of chemotherapy*	Healthy after 24 months of follow‐up	IHC: EBNA1+/BZLF1–	Yes (100%)	t(8;14) break‐apart and fusion probes
Case 4	12/M	Mandibular mass	I	Six cycles of chemotherapy*	Healthy after 16 months of follow‐up	IHC: EBNA1+ (LMP1–, LMP2–, EBNA2–, BZLF1–)	Yes (100%)	t(8;14) break‐apart and fusion probes

EBNA, Epstein–Barr nuclear antigen; EBV, Epstein–Barr virus; F, female; IHC, immunohistochemistry; LMP, latent membrane protein; LYWS, Lymphoma Workshop; M, male.

* Rituximab plus hyperfractionated cyclophosphamide, vincristine, doxorubicin, and dexamethasone, followed by methotrexate and cytarabine.

**Figure 1:**
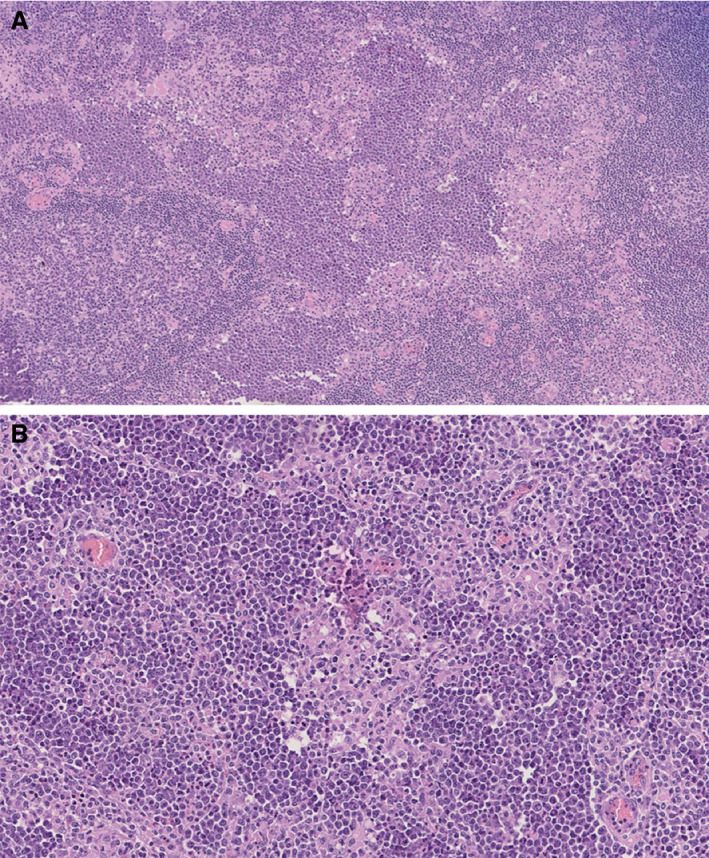
Morphology of case 1. **A,** Nests of dark‐appearing atypical cells were occasionally present in the marginal zone of the otherwise reactive lymph node. **B,** Small to medium‐sized lymphoid cells with round nuclei, finely clumped chromatin, multiple small peripheral nucleoli, surrounded by a granulomatous epithelioid reaction.

**Figure 2:**
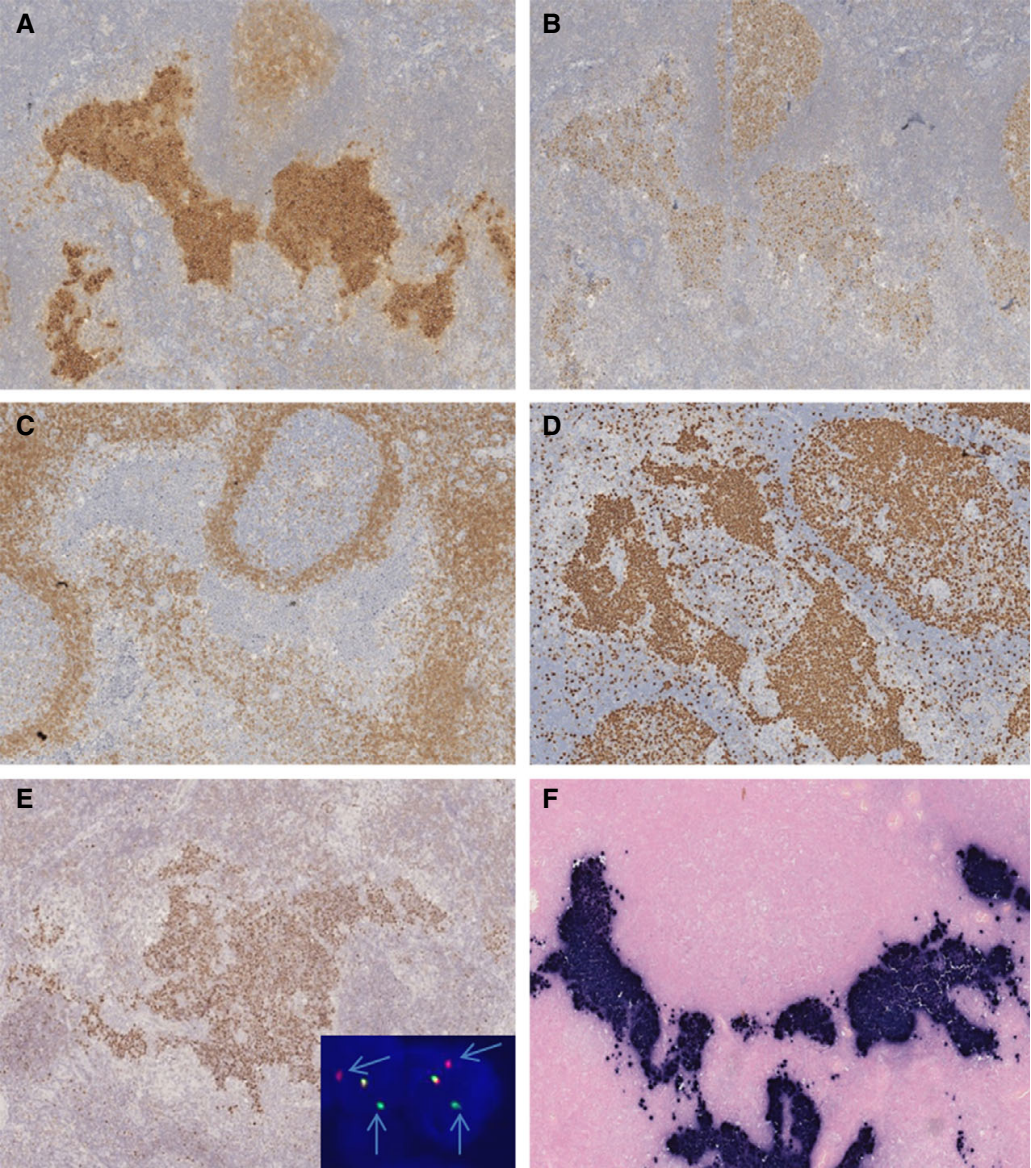
Immunophenotype of case 1. The immunophenotype of the atypical cells, i.e. CD10 positivity (**A**), bcl‐6 positivity (**B**), bcl‐2 negativity (**C**), a high Ki67 proliferation index of >95% (**D**), and MYC protein expression (**E)** and cytogenetics (*MYC* rearrangement as determined with break‐apart probes; **E,** inset), was consistent with Burkitt lymphoma. These cells were also EBV‐positive as determined with Epstein–Barr virus‐encoded small RNA *in‐situ* hybridisation (**F**).

In particular, in case 1, morphological examination of the surgical specimens revealed an overall preserved lymph node architecture characterised by follicular hyperplasia with reactive germinal centres (GCs), and prominent perisinusoidal and perifollicular clear areas composed of monocytoid B‐lymphoid cells. Exclusively within the monocytoid B‐lymphoid cell areas, nests of dark‐appearing, atypical cells were occasionally present (Figure 1A). Some nests appeared to coalesce focally, forming wider ill‐defined aggregates composed of medium‐sized lymphoid cells with round nuclei, finely clumped chromatin, multiple small peripheral nucleoli, and small to moderate amounts of basophilic cytoplasm, morphologically consistent with BL cells (Figure 1B). Mitotic figures and apoptotic bodies were seen within the nests and focal ill‐defined aggregates. In addition, a florid granulomatous reaction, sometimes encircling the neoplastic clusters of cells, was present (Figure 1B; haematoxylin and eosin‐ HE). The immunophenotype (CD20, CD10, bcl‐6 and IgM positivity, bcl‐2 negativity, a high Ki67 proliferative index of >95%) (Figure 2A–D; Table 1), MYC protein expression (Figure 2E), and cytogenetics (*MYC* translocation as shown with both dual fusion and a BAP; Figure 2E, inset) were consistent with BL (Table 1). The tumour cells were also EBV‐positive as shown by EBER *in‐situ* hybridisation (Figure 2F). Immunohistochemistry (IHC) revealed only the expression of EBNA1 among EBV latency proteins, whereas latent membrane protein 1 (LMP1), latent membrane protein (LMP2), EBNA2 and BZLF1 were negative (Table 1). Therefore, the diagnosis of focal involvement by EBV+ BL in a background of reactive lymphadenitis was made.

**Figure 3:**
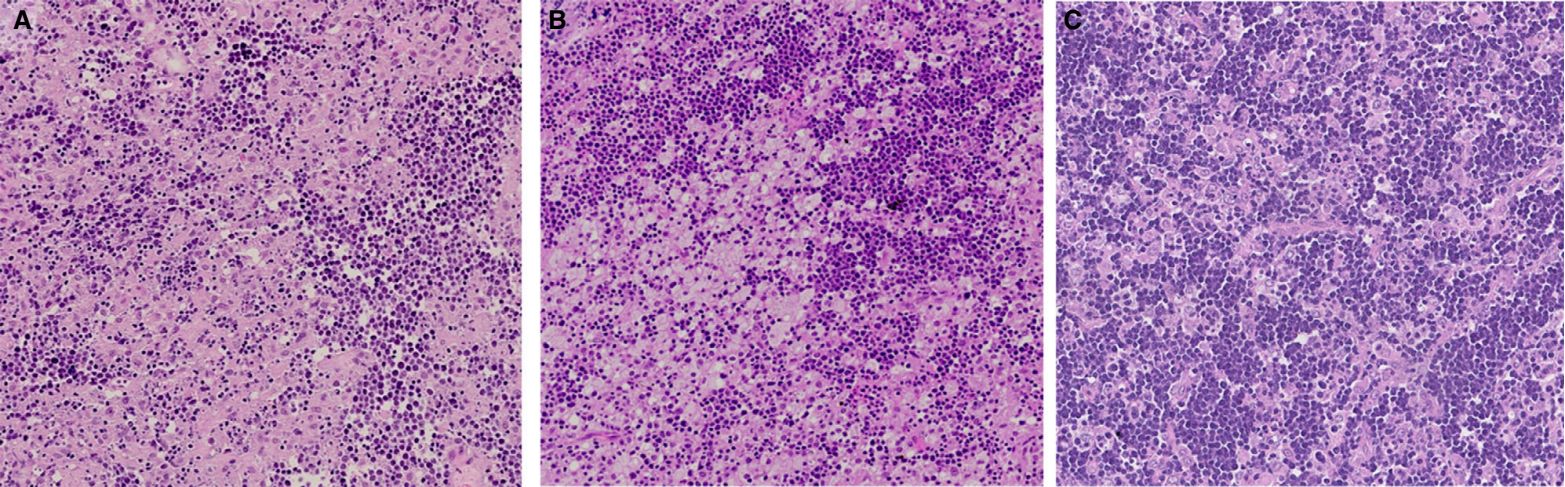
Morphological features of case 2, case 3, and case 4. There was a prominent granulomatous reaction, comprising epithelioid histiocytes and fibroblasts surrounding Burkitt lymphoma cells, in case 2 (**A**), case 3 (**B**), and case 4 (**C**).

Cases 2, 3 and 4 (Figure 3) showed a clearly effaced lymph node architecture with the presence of an overt BL with a florid granulomatous reaction, in contrast to the limited and interfollicular pattern found in case 1.

Moreover, case 2 was characterised by the presence of patchy BL cells intermingled with histiocytes, fibroblasts, and abundant collagen deposition. On the other hand, case 3 showed extensive necrotic areas with conspicuous foci of apoptotic debris.

### TME FEATURES

**Figure 4:**
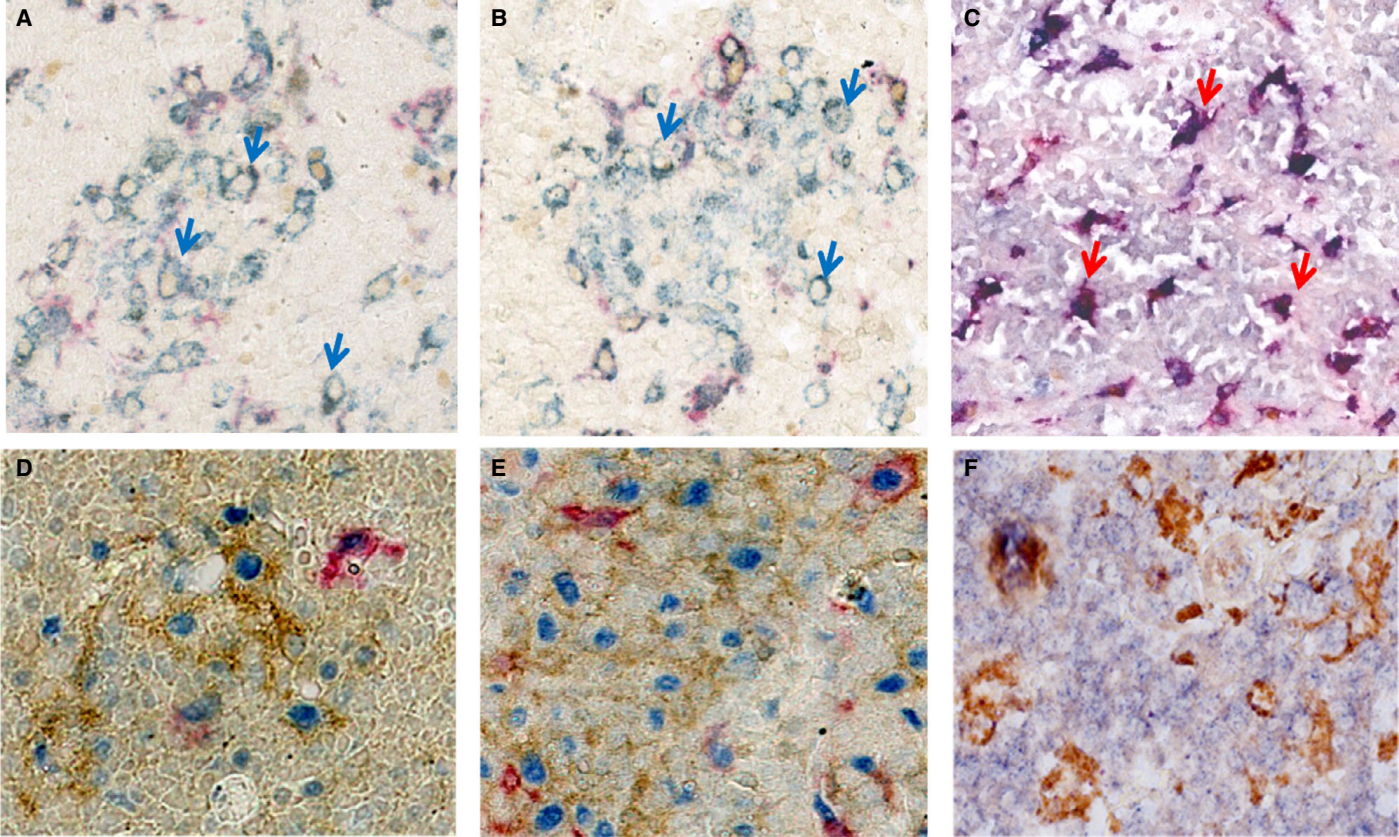
Immunohistochemical characterisation of macrophages. Triple staining was performed with c‐Maf (brown), CD163 (red), CD68 (blue), phosphorylated signal transducer and activator 1 (pSTAT1) (blue), CD68 (brown), and CD123 (red). A high number of M1 (CD68+/CD163–/c‐Maf–) macrophages (blue arrows) were seen in the granulomas surrounding the clusters of Burkitt cells in case 1 (**A**) and case 2 (**B**); in contrast, in the conventional Burkitt lymphoma (BL) case control (**C**), the predominant macrophage population was represented by M2 (CD68+/CD163+/c‐Maf+) macrophages (red arrows). Interferon‐γ‐primed macrophages and plasmacytoid dendritic cells expressed nuclear pSTAT1 in case 1 (**D**) and case 2 (**E**). On the other hand, only a few CD123+/pSTAT1+ cells were found in the conventional BL case control (**F**).

Analysis of TME in all four cases showed comparable characteristics Overall, in all four cases, analysis of the macrophages encircling the tumour areas by the use of mIHC showed a prevalence of M1 macrophages, defined as CD68+/CD163–/c‐Maf– cells, accounting for 80–95% of the total macrophages, whereas M2 macrophages (CD68+/CD163+/c‐Maf+) were scarce (5–20%) (Figure 4A,B; Table 2). In the reactive lymph nodes, the plasticity of M1 and M2 may be more active in controlling the immune responses to different antigens.^26^, ^27^ Most of the macrophages in GCs are M2, and this is possibly the reason for the high number of M2 macrophages that we found in reactive lymph nodes (personal communication of C. Tripodo, Human Pathology Section, Department of Health Sciences, University of Palermo; Figure S1).

**Table 2:** Tumour microenvironment results

Cell populations	Case 1 (%)	Case 2 (%)	Case 3 (%)	Case 4 (%)	Control (%)
Macrophages					
M1 (CD68+/CD163–/c‐Maf–)	95	85	90	80	25–35
M2 (CD68+/CD163+/c‐Maf+)	5	15	10	20	55–70
pSTAT1+ DCs					
CD68+/pSTAT1+/CD123–	65	75	40	35	<5
CD68–/pSTAT1+/CD123+	35	20	15	15	<5
T lymphocytes					
CD4+	90	80	85	75	50–70
Th1 (TBX21+/CD4+)	35	20	30	25	5–10
Th2 (GATA3+/CD4+)	10	15	25	15	25–40
Tregs (CD25+/CD4+/FOXP3+)	<5	<5	<5	<5	5–15
Cytotoxic (CD8+)	15	20	15	25	30–40
NK cells					
CD56+/CD57+	<5	<5	<5	<5	<5

DC, dendrtitic cell; FOXP3, forkhead box P3; GATA3, GATA‐binding protein 3; NK, natural killer; pSTAT1, phosphorylated signal transducer and activator 1; TBX21, T‐box transcription factor 21.

**Figure 5:**
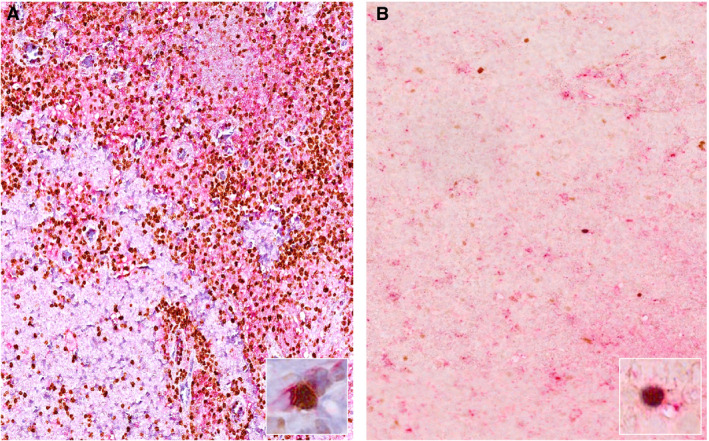
Characterisation of a Th1 infiltrate with CD4/T‐box protein expressed in T cells (T‐bet) double staining (CD4, red; T‐bet, brown). A reactive lymphoid infiltrate, mostly composed of CD4+ T cells, in particular Th1 cells (CD4+/T‐bet+), is shown; this was found in the immediate proximity of the granulomas in case 1 (**A**,**A** inset: higher‐magnification double‐positive stains); on the other hand, only a few scattered Th1 cells were detected in the conventional Burkitt lymphoma case control (**B,B** inset).

The pSTAT1/CD68/CD123 triple staining highlighted that macrophages (CD68+) express nuclear pSTAT1, indicating IFN‐γ priming and confirming an M1 phenotype of the macrophages (Figure 4D,E; Table 2). The proportion of IFN‐γ‐primed macrophages (CD68+/pSTAT1+) ranged from 35% to 75% of the total CD68+ cells, in contrast to the considerably lower proportion detected in the controls (<5%). In addition, pSTAT1 nuclear staining was also found in scattered and small clusters of CD123+ cells, in the proximity of granulomas, possibly representing activated pDCs, and ranging from 15% to 35% (Figure 4A,B; Table 2). On the other hand, only a few CD123+/pSTAT1+ cells were found in the controls (<5%). In addition, the PD‐L1/CD163/c‐Maf panel showed that epithelioid granulomas were significantly positive for PD‐L1, as a secondary effect of IFN‐γ activation (Figure S2). The dominant reactive lymphoid infiltrate was mostly composed of CD4+ T cells, ranging from 75% to 90% of the total CD4+ T cells. In particular, Th1 cells (CD4+/T‐bet+ cells) were consistently represented, ranging from 20% to 35% of the total CD4+ T cells (Figure 5A; Table 2). Interestingly, Th1 lymphocytes were found in the immediate proximity of the granulomas, further underlining the cooperation between innate and adaptive immunity. In contrast, the tumour‐free areas and the reactive lymph node control showed a scant or absent Th1 infiltrate (5–10%) (Figure S3A; Table 2). However, CD4/T‐bet double staining revealed a subpopulation of CD4–/T‐bet+ cells. Double staining with CD20 showed the B‐cell origin of such cells, which more probably represented a subset of monocytoid B cells, as previously reported by Jöhrens *et al*.^26^ This finding was particularly evident in case 1, in which BL cells were nested in areas of florid monocytoid B‐cell hyperplasia (Figure S4), but was also made in the other cases, maintaining the topographic correlation with the granulomas. Th2 cells (CD4+/GATA3+ cells) were also represented in the tumour areas, ranging from 10% to 25%. However, these values were lower than those in the reactive tissue (Figure S3B; Table 2). A few scattered cytotoxic CD8+ T cells (<5%; Table 2), Tregs (CD4+/CD25+/FOXP3+; <5%; Table 2) and NK cells (CD56+/CD57+; <5%; Table 2) were seen (not shown).

## Discussion

**Figure 6:**
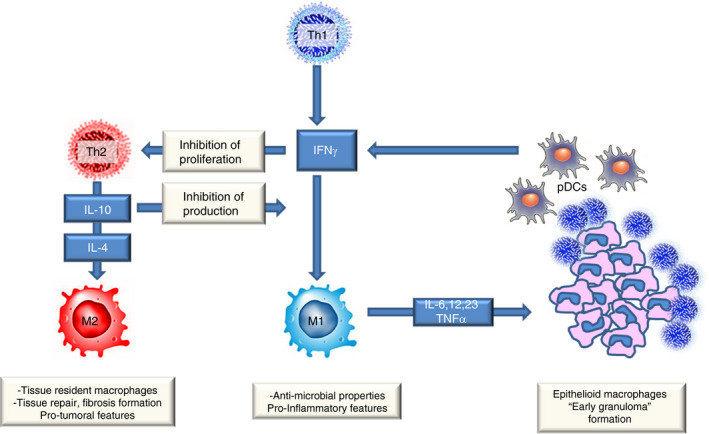
The role of Th1 lymphocytes. Th1 lymphocytes are well‐known interferon‐γ‐secreting cells that activate inflammatory pathways, mainly via macrophage polarisation towards an M1 functional status. In addition, they promote granuloma formation and inhibit Th2 lymphocyte proliferation.

In this article, we report four cases of localised (stage I) EBV‐positive BL with a prominent granulomatous reaction and a similar TME consisting of Th1 lymphocytes and M1 macrophages. The TME described above is quite different from that of conventional BL with the typical starry sky pattern, in which the most prominent component of TME is represented by M2 macrophages.^23^ Such cases usually present with bulky disease and multiple localisation, and intensive chemotherapy is required. On the other hand, cases of BL with a florid granulomatous reaction typically present at an early stage of disease and have a particularly good prognosis, with some cases showing spontaneous regression.^1^, ^2^, ^3^, ^4^, ^5^, ^6^ Although a proinflammatory immune reaction may be responsible for the favourable outcome, thorough characterisation of the reactive infiltrate in these cases was lacking. Using mIHC, we have now shown that the TME has features of an activated cellular immune response characterised by the prevalence of Th1 lymphocytes and M1 macrophages forming granulomas enclosing neoplastic cells. Th1 lymphocytes are well‐known IFN‐γ‐secreting cells that activate inflammatory pathways mainly via macrophage polarisation towards an M1 functional status. In addition, they promote granuloma formation and inhibit Th2 lymphocyte proliferation (Figure 6).^9^, ^11^ Accordingly, the vast majority of macrophages were primed by IFN‐γ, as demonstrated by pSTAT1 nuclear positivity.^9^, ^10^ In addition, because of the dichotomous role of IFN‐γ, which activates both the immune innate response and on the checkpoint inhibitors, the positivity for PD‐L1 in the granulomas’ epithelioid macrophages may indicate, in our context, strong activation of the innate immune response.^28^, ^29^

Another important observation in this study was the peculiar topographic distribution of the reactive inflammatory cells. In fact, Th1 lymphocytes, pDCs and M1 macrophages were detected in the immediate proximity of the BL cells. Intriguingly, we also observed numerous CD20+/T‐bet+ cells, which more probably represented monocytoid B cells, around the clusters of BL cells.^30^ There are recent data suggesting that T‐bet is a central regulator of antiviral immunity in all lymphocyte lineages, and that T‐bet+ B cells may reresent an important component of ongoing immune responses during chronic viral infections.^31^ Furthermore, our results are in accordance with previous observations that have underlined the role of EBNA1‐specific CD4+ T cells in the regulation of the immune response in healthy carriers of EBV.^17^, ^18^, ^19^, ^20^, ^21^ Our data may well represent an *in‐vivo* picture of a T‐bet‐mediated immune response fostered by IFN‐γ and subsequent granuloma formation comprising M1 polarised macrophages with an activated STAT1 signalling pathway.

We believe that the fractions of cells that we scored in our IHC experiments reflect specific Th states according to the expression of selected Th proteins. However, we need to consider a variety of states and transitions that may form part of the highly dynamic tumour‐infiltrating T‐cell landscape and that we are not taking into account. Besides Th1 and Th2, in addition Th0, Th17, Th9 and Th22 cells and other potentially relevant Th phenotypes could contribute to the overall Th infiltration of BL, such as bcl‐6+ and/or c‐Maf+ T follicular helper cells, which are variably represented among CD4+ T cells in lymphomas of the GC phenotype, or variants in regulatory subsets that may be aberrantly expanded in tumours, such as eomesodermin+/granzyme K+/type 1 regulatory‐like CD4+ effectors.^32^

Therefore, our findings need to be confirmed in further studies using different methods that allow more comprehensive characterisation of the TME (e.g. gene expression analysis).

The interplay with the TME may be a crucial event for the outcome of EBV‐related diseases, and the viral interaction with host immune surveillance requires further investigation in correlation with the EBV latency programme. It has been shown that the pattern of viral protein expression determines the immunogenicity of the infected cells.^33^, ^34^ The virus encodes eight antigenically distinct latent‐cycle proteins, which show a marked hierarchy of immunodominance for the CD8+/CD4+ T‐cell response. Epitopes derived from the EBNA3A, EBNA3B and EBNA3C family of proteins tend to induce the strongest responses across a range of different HLA class I alleles. LMP1‐specific responses are extremely rare, whereas LMP2 is more frequently immunogenic but almost always induces low‐frequency subdominant responses.^35^ Although EBNA1 was initially supposed to mount an immunologically weak response, more recent studies demonstrated that healthy EBV carriers had an efficient CD4+ T cell response to this antigen that was mainly Th1 in nature.^36^, ^37^ On these grounds, conventional BLs with EBV latency I may show a different immune signature from those with a heterogeneous latency programme characterised by the expression of LMP1, LMP2, and/or lytic genes (non‐canonical latency programme).^23^, ^38^, ^39^ Indeed, all of the cases reported here showed a latency I programme, with the sole expression of EBNA1.

A further observation of this study concerned the distinctive morphological features of case 1. In fact, in this case the nests of BL cells were found exclusively within prominent areas of clear cells identified as monocytoid B cells, which might represent a possible niche from which BL cells originate and spread, as has been previously reported for Hodgkin’s lymphoma.^40^, ^41^ Although there is consensus that BL is related to GC B lymphocytes, it has been hypothesised that EBV‐positive BL cases may derive from a later developmental stage of B cells, i.e. post‐GC/memory B cells.^42^, ^43^ This is in line with the fact that, in healthy carriers, EBV resides in memory B cells that re‐enter the GC reaction following antigenic stimulation.^44^, ^45^, ^46^ Only one case with similar features has been recently reported in the literature, in a human immunodeficiency virus (HIV)‐positive female,^47^ and was reported as ‘Burkitt microlymphoma’. We have also observed a similar case in an HIV‐positive patient (Figure S5). Both cases developed overt BL with an unfavourable outcome shortly after the diagnosis, whereas the case reported here occurred in an immunocompetent host, and was characterised by a granulomatous reaction and spontaneous regression, possibly representing an early phase of BL.

BL in the setting of a granulomatous reaction and conspicuous monocytoid B‐cell hyperplasia might be easily overlooked, and making the correct diagnosis may be challenging.^6^ Awareness of these features is important to avoid misdiagnosis. Our study provides, for the first time, an *in‐vivo* picture of the immune surveillance of BL with a granulomatous reaction that might explain why an aggressive lymphoma is kept in check and behaves in such a self‐limiting way. In contrast to conventional BL, the important role of Th1 and M1 macrophages in the TME is highlighted. In addition, the recognition of TME features of BL may be helpful for identifying a subset of BL patients with a better prognosis, and who are thus suitable for less burdensome therapy. Further studies are warranted to investigate the reason why similar tumour cells trigger different immune responses.

In addition, our data may provide the rationale for new potential therapeutic avenues to explore in EBV‐positive BL patients in the era of immunotherapy, and in particular adoptive T‐cell therapy with EBNA1‐specific Th1 cells.^48^, ^49^

## Conflicts of interest

The authors declare that they have no conflicts of interest.

## Compliance with ethical standards

All procedures performed in studies involving human participants were in accordance with the ethical standards of the institutional research committee and with the 1964 Helsinki declaration and its later amendments or comparable ethical standards. This was a non‐interventional study on archived tissue samples.

## Author contributions

R. Santi, F. Vergoni, D. Di Stefano, B. Puccini, E. Sabattini, C. Agostinelli, N. Bassüllü, T. Tecimer, A. S. Demiroz, L. Mnango, and S. Dirnhofer: provided tumour samples and clinical data. A. Akarca, E. Sorrentino, R. Guazzo, and L. Mundo: performed IHC and cytogenetic analysis. M. Granai, S. Lazzi, V. Mancini, R. Santi, F. Vergoni, G. Cevenini, C. Tripodo, G. Di Stefano, M. Ponzoni, E. Sabattini, C. Agostinelli, S. Dirnhofer, T. Marafioti, L. Quintanilla‐Martinez, F. Fend, and L. Leoncini: analysed and interpreted the data. M. Granai, S. Lazzi, T. Marafioti, L. Quintanilla‐Martinez, F. Fend, and L. Leoncini: designed and coordinated the study. M. Granai, S. Lazzi, L. Quintanilla‐Martinez, F. Fend, and L. Leoncini: interpreted the data and wrote the manuscript.

## Supporting information


**Data S1**. Supplementary materials and methods.

**Figure S1**. High number of M2 macrophages in a reactive lymph node.

**Figure S2**. Dichotomous role of IFN‐γ.

**Figure S3**. Th1 and Th2 expression in the controls.

**Figure S4**. Expression of T‐bet in B cells.

**Figure S5**. Partial lymph node involvement by BL in an HIV‐positive patient.

**Table S1**. Antibody characteristics.

**Table S2**. The individual scores of the observers and the agreement for all separate cell types.
