# Supplementary material for: Burkitt lymphoma with a granulomatous reaction: an M1/Th1‐polarised microenvironment is associated with controlled growth and spontaneous regression
Source: Histopathology. 2021 Jul 5;80(2):430–42. doi: 10.1111/his.14391 (PMC9291779; doi:10.1111/his.14391)
Supplement: Supplementary file 1 — Data S1. Supplementary materials and methods. [file HIS-80-430-s002.docx]

1. [ version 19 ]
2. [ For Research Use Only. Not intended for diagnostic purposes. ]
3. Enable mixer
4. Heat slides at37°C
5. [ Delay refers to a time delayed start: Select time until run start ]
6. Disable mixer
7. [ 72°C is standard temperature ]
8. Heat slide at [72°C] from medium Temperature ( Dewaxing )
9. Incubate 4 minutes
10. Fix EZ Prep volume(Discovery)
11. Rinse slide with EZ Prep
12. Fix EZ Prep volume(Discovery)
13. Dispense Coverslip
14. Rinse slide with EZ Prep
15. Fix EZ Prep volume(Discovery)
16. Dispense Coverslip
17. Enable mixer
18. Heat slides at 37°C
19. Rinse slide with EZ Prep
20. Dispense Cell Conditioner long 1
21. Dispense Coverslip per CC long
22. Heat slides at[100°C] and incubate 4 minutes (Cell Conditioner 1)
23. Incubate 4 minutes
24. Incubate 8 minutes
25. Dispense Cell Conditioner 1
26. Dispense CC Coverslip medium, without Bar Code Blowoff
27. Incubate 8 minutes
28. Incubate 8 minutes
29. Dispense Cell Conditioner 1
30. Dispense CC Coverslip medium, without Bar Code Blowoff
31. Incubate 8 minutes
32. Incubate 8 minutes
33. Dispense Cell Conditioner 1
34. Dispense CC Coverslip medium, without Bar Code Blowoff
35. Incubate 8 minutes
36. Dispense Cell Conditioner 1
37. Dispense CC Coverslip medium, without Bar Code Blowoff
38. Dispense Cell Conditioner 1
39. Dispense CC Coverslip medium, without Bar Code Blowoff
40. Dispense Cell Conditioner 1
41. Dispense CC Coverslip medium, without Bar Code Blowoff
42. Disable slides heating
43. Dispense Cell Conditioner 1
44. Dispense CC Coverslip medium, without Bar Code Blowoff
45. Heat slides at37°C

- One drop equals one aliquot of reagent Stampato 22/02/2021 10:43:23 Ventana Medical Systems, Inc., 1910 Innovation Park Drive Tucson, Arizona USA Pagina 1 di 4 VSS v12.4 Build 15110.1

1. Rinse slide with Reaction Buffer
2. Fix volume on the slide with Reaction Buffer
3. Dispense Coverslip
4. [ Select an Inhibitor ]
5. [ NOTE: Inhibitor CM comes packaged with Chromomap DAB; InhibitorD comes packaged with DABMap ]
6. [ DISCOVERY Inhibitor is a stand alone product for use with all other HRP substrates ]
7. [ Inhibitor CM will be applied ]
8. Rinse slide with Reaction Buffer
9. Fix volume on the slide with Reaction Buffer
10. Dispense Coverslip
11. Rinse slide with Reaction Buffer
12. Fix volume on the slide with Reaction Buffer
13. Dispense Coverslip
14. Dispense one aliquot of Inhibitor CM, and incubate [8 minutes]
15. Rinse slide with Reaction Buffer
16. Fix volume on the slide with Reaction Buffer
17. Dispense Coverslip
18. Disable slides heating
19. Disable mixer
20. Wait For Button ( Antibody )
21. Enable mixer
22. Heat slides at37°C
23. Incubate 4 minutes
24. Rinse slide with Reaction Buffer
25. Fix volume on the slide with Reaction Buffer
26. Dispense Coverslip
27. Incubate 4 minutes
28. Heat slide at [37°C] from very low Temperature (primary Antibody)
29. Hand Apply ( Primary Antibody T-BET VENTANA ROCHE), and Incubate for [60 minutes]
30. Rinse slide with Reaction Buffer
31. Fix volume on the slide with Reaction Buffer
32. Dispense Coverslip
33. [ Inhibitor Solution will not be applied after the primary ]
34. Disable slides heating
35. Heat slides at37°C
36. [ Requires DETECTION dispensers ]
37. [ These selections may be used for haptenated linking antibodies ]
38. Rinse slide with Reaction Buffer
39. Fix volume on the slide with Reaction Buffer
40. Dispense Coverslip
41. Rinse slide with Reaction Buffer
42. Fix volume on the slide with Reaction Buffer
43. Dispense Coverslip
44. Rinse slide with Reaction Buffer
45. Fix volume on the slide with Reaction Buffer

- One drop equals one aliquot of reagent Stampato 22/02/2021 10:43:24 Ventana Medical Systems, Inc., 1910 Innovation Park Drive Tucson, Arizona USA Pagina 2 di 4 VSS v12.4 Build 15110.1

1. Dispense Coverslip
2. Incubate 4 minutes
3. Heat slide at [36 °C] from very low Temperature ( 2^nd^ Antibody)
4. Dispense one aliquot of [Anti-Rabbit HQ] and incubate [0 ore 8 min]
5. Rinse slide with Reaction Buffer
6. Fix volume on the slide with Reaction Buffer
7. Dispense Coverslip
8. Disable slides heating
9. Heat slides at37°C
10. Rinse slide with Reaction Buffer
11. Fix volume on the slide with Reaction Buffer
12. Dispense Coverslip
13. Dispense one aliquot of H2O2 CM, and incubate 4 minutes
14. Dispense one aliquot of DAB CM, and incubate 8 minutes
15. Rinse slide with Reaction Buffer
16. Fix volume on the slide with Reaction Buffer
17. Dispense one aliquot of Copper CM, add Coverslip and incubate 4 minutes
18. Rinse slide with Reaction Buffer
19. Fix volume on the slide with Reaction Buffer
20. Dispense Coverslip
21. [ DISC Inhibitor will not be applied ]
22. Rinse slide with Reaction Buffer
23. Fix volume on the slide with Reaction Buffer
24. Dispense Coverslip
25. Disable slides heating
26. Disable mixer
27. Wait For Button (primary Antibody - (II) )
28. Enable mixer
29. Heat slides at 37°C
30. Rinse slide with Reaction Buffer
31. Fix volume on the slide with Reaction Buffer
32. Dispense Coverslip
33. Heat slide at [35 °C] from very low Temperature ( primary Antibody - (II) )
34. Hand Apply ( primary Antibody - (II) CD4 VENATA ROCHE), and incubate for [60 minutes]
35. Rinse slide with Reaction Buffer
36. Fix volume on the slide with Reaction Buffer
37. Dispense Coverslip
38. Disable slides heating
39. Heat slides at 37°C
40. Disable slides heating
41. Heat slides at 37°C
42. Rinse slide with Reaction Buffer
43. Dispense Anti-Rabbit NP
44. Dispense DISC Naphthol, DISC Fast Red, for 16 minuti
45. Fix volume on the slide with Reaction Buffer
46. Dispense Coverslip
47. Dispense one aliquot of [HEMATOXYLIN] (Counterstain) and incubate [8 minutes]

- One drop equals one aliquot of reagent Stampato 22/02/2021 10:43:24 Ventana Medical Systems, Inc., 1910 Innovation Park Drive Tucson, Arizona USA Pagina 3 di 4 VSS v12.4 Build 15110.1

1. Rinse slide with Reaction Buffer
2. Fix volume on the slide with Reaction Buffer
3. Dispense Coverslip
4. Rinse slide with Reaction Buffer
5. Fix volume on the slide with Reaction Buffer

- One drop equals one aliquot of reagent Stampato 22/02/2021 10:43:24 Ventana Medical Systems, Inc., 1910 Innovation Park Drive Tucson, Arizona USA Pagina 4 di 4 VSS v12.4 Build 15110.1

1. [ version 19 ]
2. [ For Research Use Only. Not intended for diagnostic purposes. ]
3. Enable mixer
4. Heat slides at 37°C
5. [ Delay refers to a time delayed start: Select time until run start ]
6. Disable mixer
7. [ 72°C is standard temperature ]
8. Heat slide a [72°C] from medium Temperature ( Dewaxing )
9. Incubate 4 minutes
10. Fix EZ Prep volume(Discovery) 11
11. Fix EZ Prep volume(Discovery)
12. Dispense Coverslip
13. Rinse slide with EZ Prep
14. Fix EZ Prep volume(Discovery)
15. Dispense Coverslip
16. Enable mixer
17. Heat slides at 37°C
18. Rinse slide with EZ Prep
19. Dispense Cell Conditioner l1
20. Dispense Coverslip per CC long
21. Heat slides at [100°C] and incubate 4 minutes (Cell Conditioner 1)
22. Incubate 4 minutes
23. Incubate 8 minutes
24. Dispense Cell Conditioner 1
25. Dispense CC Coverslip medium, without Bar Code Blowoff
26. Incubate 8 minutes
27. Incubate 8 minutes
28. Dispense Cell Conditioner 1
29. Dispense CC Coverslip medium, without Bar Code Blowoff
30. Incubate 8 minutes
31. Incubate 8 minutes
32. Dispense Cell Conditioner 1
33. Dispense CC Coverslip medium, without Bar Code Blowoff
34. Incubate 8 minutes
35. Dispense Cell Conditioner 1
36. Dispense CC Coverslip medium, without Bar Code Blowoff
37. Dispense Cell Conditioner 1
38. Dispense CC Coverslip medium, without Bar Code Blowoff
39. Dispense Cell Conditioner 1
40. Dispense CC Coverslip medium, without Bar Code Blowoff
41. Disable slides heating
42. Dispense Cell Conditioner 1
43. Dispense CC Coverslip medium, without Bar Code Blowoff
44. Heat slides at 37°C
45. Rinse slide with Reaction Buffer
46. Fix volume on the slide with Reaction Buffer
47. Dispense Coverslip
48. [ Select an Inhibitor ]
49. [ NOTE: Inhibitor CM comes packaged with Chromomap DAB; InhibitorD comes packaged with DABMap ]
50. [ DISCOVERY Inhibitor is a stand alone product for use with all other HRP substrates ]
51. [ Inhibitor CM will be applied ]
52. Rinse slide with Reaction Buffer
53. Fix volume on the slide with Reaction Buffer
54. Dispense Coverslip
55. Rinse slide with Reaction Buffer
56. Fix volume on the slide with Reaction Buffer
57. Dispense Coverslip
58. Dispense one aliquota of Inhibitor CM, then incubate [8 minutes]
59. Rinse slide with Reaction Buffer
60. Fix volume on the slide with Reaction Buffer
61. Dispense Coverslip
62. Disable slides heating
63. Disable mixer
64. Wait For Button
65. Enable mixer
66. Heat slides at 37°C
67. Incubate 4 minutes
68. Rinse slide with Reaction Buffer
69. Fix volume on the slide with Reaction Buffer
70. Dispense Coverslip
71. Incubate 4 minutes
72. Heat slide a [37°C] from very low Temperature ( Primary Antibody )
73. Hand Apply ( Primary Antibody GATA 3 VENTANA ROCHE L50-823), and Incubate for [60 minutes]
74. Rinse slide with Reaction Buffer
75. Fix volume on the slide with Reaction Buffer
76. Dispense Coverslip
77. [ Inhibitor Solution will not be applied after the primary ]
78. Disable slides heating
79. Heat slides at 37°C
80. [ Requires DETECTION dispensers ]
81. [ These selections may be used for haptenated linking antibodies ]
82. Rinse slide with Reaction Buffer
83. Fix volume on the slide with Reaction Buffer
84. Dispense Coverslip
85. Rinse slide with Reaction Buffer
86. Fix volume on the slide with Reaction Buffer
87. Dispense Coverslip
88. Rinse slide with Reaction Buffer
89. Fix volume on the slide with Reaction Buffer
90. Dispense Coverslip
91. Incubate 4 minutes
92. Heat slide a [36 °C] from very low Temperature ( 2nd Antibody)
93. Dispense one aliquot of [Anti-Mouse HQ] (Revelation 1) and incubate [0 ore 8 min]
94. Rinse slide with Reaction Buffer
95. Fix volume on the slide with Reaction Buffer
96. Dispense Coverslip
97. Disable slides heating
98. Heat slides at 37°C
99. Rinse slide with Reaction Buffer
100. Fix volume on the slide with Reaction Buffer
101. Dispense Coverslip
102. Dispense one aliquot H2O2 CM, and incubate 4 minutes
103. Dispense one aliquot of DAB CM, and incubate 8 minutes
104. Rinse slide with Reaction Buffer
105. Fix volume on the slide with Reaction Buffer
106. Dispense one aliquot of Copper CM, add Coverslip and incubate 4 minutes
107. Rinse slide with Reaction Buffer
108. Fix volume on the slide with Reaction Buffer
109. Dispense Coverslip
110. [ DISC Inhibitor will not be applied ]
111. Rinse slide with Reaction Buffer
112. Fix volume on the slide with Reaction Buffer
113. Dispense Coverslip
114. Disable slides heating
115. Disable mixer
116. Wait For Button ( Primary Antibody - (II) )
117. Enable mixer
118. Heat slides at 37°C
119. Rinse slide with Reaction Buffer
120. Fix volume on the slide with Reaction Buffer
121. Dispense Coverslip
122. Heat slide a [35 °C] from very low Temperature ( Primary Antibody - (II) )
123. Hand Apply ( Primary Antibody -CD4 VENATANA ROCHE SP35 (II) ), and Incubate for [60 minutes]
124. Rinse slide with Reaction Buffer
125. Fix volume on the slide with Reaction Buffer
126. Dispense Coverslip
127. Disable slides heating
128. Heat slides at 37°C
129. Disable slides heating
130. Heat slides at 37°C
131. Rinse slide with Reaction Buffer

134 Dispense [Anti-Rabbit NP] (Rivelazione 4) for 0 h 8 min

1. Dispense Anti-NP AP
2. Dispense DISC Naphthol, DISC Fast Red, for 16 minuti
3. Dispense Coverslip
4. Dispense one aliquot of [HEMATOXYLIN] (Counterstain) and incubate [8 minutes]

- One drop equals one aliquot of reagent Stampato 22/02/2021 10:32:53 Ventana Medical Systems, Inc., 1910 Innovation Park Drive Tucson, Arizona USA Pagina 3 di 4 VSS v12.4 Build 15110.1

1. Rinse slide with Reaction Buffer
2. Fix volume on the slide with Reaction Buffer
3. Dispense Coverslip
4. Rinse slide with Reaction Buffer

- One drop equals one aliquot of reagent Stampato 22/02/2021 10:32:53 Ventana Medical Systems, Inc., 1910 Innovation Park Drive Tucson, Arizona USA Pagina 4 di 4 VSS v12.4 Build 15110.1

1. [ version 19 ]
2. [ For Research Use Only. Not intended for diagnostic purposes. ]
3. Enable mixer
4. Heat slides at 37°C
5. [ Delay refers to a time delayed start: Select time until run start ]
6. Disable mixer
7. [ 72°C is standard temperature]
8. Heat slide at [72°C] from Medium Temperature ( Dewaxing )
9. Incubate 4 minutes.
10. Fix EZ Prep volume (Discovery)
11. Rinse slide with EZ Prep
12. Fix EZ Prep volume (Discovery)
13. Dispense Coverslip
14. Rinse slide with EZ Prep
15. Fix EZ Prep volume (Discovery)
16. Dispense Coverslip
17. Enable mixer
18. Heat slides at 37°C
19. Rinse slide with EZ Prep
20. Dispense Cell Conditioner
21. Dispense Coverslip per CC
22. Heat slides [100°C] and incubate 4 minutes (Cell Conditioner 1)
23. Incubate 4 minutes
24. Incubate 8 minutes
25. Dispense Cell Conditioner 1
26. Dispense CC Coverslip medium, without Bar Code Blowoff
27. Incubate 8 minutes
28. Incubate 8 minutes
29. Dispense Cell Conditioner 1
30. Dispense CC medium Coverslip, senza Bar Code Blowoff
31. Incubate 8 minutes
32. Incubate 8 minutes
33. Dispense Cell Conditioner 1
34. Dispense CC medium Coverslip, without Bar Code Blowoff
35. Incubate 8 minutes
36. Dispense Cell Conditioner 1
37. Dispense CC medium Coverslip, without Bar Code Blowoff
38. Dispense Cell Conditioner 1
39. Dispense CC medium Coverslip, without Bar Code Blowoff
40. Dispense Cell Conditioner 1
41. Dispense CC medium Coverslip, without Bar Code Blowoff
42. Disable slides heating
43. Dispense Cell Conditioner 1
44. Dispense medium CC Coverslip, without Bar Code Blowoff
45. Heat slides 37°C
46. Rinse slide with Reaction Buffer
47. Fix volum on the slide with Reaction Buffer
48. Dispense Coverslip
49. [ Select an Inhibitor ]
50. [ NOTE: Inhibitor CM comes packaged with Chromomap DAB; InhibitorD comes packaged with DABMap ]
51. [ DISCOVERY Inhibitor is a stand alone product for use with all other HRP substrates ]
52. [ Inhibitor CM will be applied ]
53. Rinse slide with Reaction Buffer
54. Fix volume on the slide with Reaction Buffer
55. Dispense Coverslip
56. Rinse slide with Reaction Buffer
57. Fix volume on the slide with Reaction Buffer
58. Dispense Coverslip
59. Dispense one Inhibitor CM aliquot, then incubate [8 minutes]
60. Rinse slide with Reaction Buffer
61. Fix volume on the slide with Reaction Buffer
62. Dispense Coverslip
63. Disable slide heating
64. Disable mixer
65. Wait For Button ( Antibody )
66. Enable mixer
67. Heat slides at 37°C
68. Incubate 4 minutes
69. Rinse slide with Reaction Buffer
70. Fix volume on the slide with Reaction Buffer
71. Dispense Coverslip
72. Incubate 4 minutes
73. Heat slide [60°C] from Medium Temperature ( Primary Antibody )
74. Hand Apply ( Primary Antibody p-Stat 1 ABCAM), and Incubate for [32 minutes]
75. Rinse slide with Reaction Buffer
76. Fix volume on the slide with Reaction Buffer
77. Dispense Coverslip
78. [ Inhibitor Solution will not be applied after the primary ]
79. Disableil slides heating
80. Heat slides at 37°C
81. [ Requires DETECTION dispensers ]
82. [ These selections may be used for haptenated linking antibodies ]
83. Rinse slide with Reaction Buffer
84. Fix volume on the slide with Reaction Buffer
85. Dispense Coverslip
86. Rinse slide with Reaction Buffer
87. Fix volume on the slide with Reaction Buffer
88. Dispense Coverslip
89. Rinse slide with Reaction Buffer
90. Fix volume on the slide with Reaction Buffer
91. Dispense Coverslip
92. Incubate 4 minutes
93. Heat slide a [36 °C] from Very Low Temperature ( Secondary Antibody )
94. Dispense one [Anti-Mouse HQ] aliquot (Revelation 1), then incubate [0 ore 8 min]
95. Rinse slide with Reaction Buffer
96. Fix volume on the slide with Reaction Buffer
97. Dispense Coverslip
98. Disable slides heating
99. Heat i slides a 37°C
100. Rinse slide with Reaction Buffer
101. Fix volume on the slide with Reaction Buffer
102. Dispense Coverslip
103. Rinse slide with Reaction Buffer
104. Fix volume on the slide with Reaction Buffer
105. Dispense Coverslip
106. [ Select Multimer ]
107. Dispense one [Anti-HQ HRP] aliquot (Conjugate #1) then incubate [8 minutes]
108. Rinse slide with Reaction Buffer
109. Fix volume on the slide with Reaction Buffer
110. Dispense Coverslip
111. Rinse slide with Reaction Buffer
112. Fix volume on the slide with Reaction Buffer
113. Dispense Coverslip
114. Rinse slide with Reaction Buffer
115. Fix volume on the slide with Reaction Buffer
116. Dispense Coverslip
117. Dispense one H2O2 CM aliquot, then incubate 4 minutes
118. Dispense one DAB CM aliquot, then incubate 8 minutes
119. Rinse slide with Reaction Buffer
120. Fix volume on the slide with Reaction Buffer
121. Dispense one Copper CM aliquot, add Coverslip then incubate 4 minutes
122. Rinse slide with Reaction Buffer
123. Fix volume on the slide with Reaction Buffer
124. Dispense Coverslip
125. [ DISC Inhibitor will not be applied ]
126. Rinse slide with Reaction Buffer
127. Fix volume on the slide with Reaction Buffer
128. Dispense Coverslip
129. Disable slides heating
130. Disable mixer
131. Wait For Button ( Primary Antibody CD163 ABCAM)
132. Enable mixer
133. Heat slides at 37°C
134. Rinse slide with Reaction Buffer
135. Fix volume on the slide with Reaction Buffer
136. Dispense Coverslip
137. Heat slide [37°C] from Very Low Temperature ( Primary Antibody - (II) )
138. Hand Apply ( Primary Antibody - (II) ), and Incubate for [36 minutes]
139. Rinse slide with Reaction Buffer
140. Fix volume on the slide with Reaction Buffer
141. Dispense Coverslip
142. Disable slides heating.
143. Heat slides at 37°C
144. [ Requires DETECTION dispensers ]
145. Rinse slide with Reaction Buffer
146. Fix volume on the slide with Reaction Buffer
147. Dispense Coverslip
148. Rinse slide with Reaction Buffer
149. Fix volume on the slide with Reaction Buffer
150. Dispense Coverslip
151. Rinse slide with Reaction Buffer
152. Fix volume on the slide with Reaction Buffer
153. Dispense Coverslip
154. Incubate 4 minutes
155. Heat slide [36 °C] from Very Low Temperature ( 2nd Antibody - (II) )
156. Dispense one [Anti-Mouse NP] aliquote (Rivelazione 4) then incubate [0 ore 8 min]
157. Rinse slide with Reaction Buffer
158. Fix volume on the slide with Reaction Buffer
159. Dispense Coverslip
160. Disaable slides heating
161. Heat slides a 37°C
162. Rinse slide with Reaction Buffer
163. Fix volume on the slide with Reaction Buffer
164. Dispense Coverslip
165. Rinse slide with Reaction Buffer
166. Fix volume on the slide with Reaction Buffer
167. Dispense Coverslip
168. [ Select Multimer ]
169. Dispense one [Anti-NP AP] aliquot (Conjugate #2) then incubate per [8 minutes]
170. Rinse slide with Reaction Buffer
171. Fix volume on the slide with Reaction Buffer
172. Dispense Coverslip
173. Rinse slide with Reaction Buffer
174. Fix volume on the slide with Reaction Buffer
175. Dispense Coverslip
176. Rinse slide with Reaction Buffer
177. Fix volume on the slide with Reaction Buffer
178. Dispense one aliquot of DISC Naphthol and one of DISC Fast Red, add Coverslip and incubate per [16 minutes
179. Rinse slide with Reaction Buffer
180. Fix volume on the slide with Reaction Buffer
181. Dispense Coverslip
182. [ DISC Inhibitor will not be applied ]
183. Rinse slide with Reaction Buffer
184. Fix volume on the slide with Reaction Buffer
185. Dispense Coverslip
186. Disable slides heating
187. Disable mixer
188. Wait For Button ( Primary Antibody - (II) )
189. Enable mixer
190. Heat slides at 37°C
191. Rinse slide with Reaction Buffer
192. Fix volume on the slide with Reaction Buffer
193. Dispense Coverslip
194. Heat slide [36 °C] from Very Low Temperatures ( TS Primary Antibody )
195. Hand Apply ( TS Primary Antibody CD68 ABCAM), and Incubate for [56 minutes]
196. Rinse slide with Reaction Buffer
197. Fix volume on the slide with Reaction Buffer
198. Dispense Coverslip
199. Disable slides heating
200. Heat slides a 37°C
201. [ Requires DETECTION dispensers ]
202. Rinse slide with Reaction Buffer
203. Fix volume on the slide with Reaction Buffer
204. Dispense Coverslip
205. Rinse slide with Reaction Buffer
206. Fix volume on the slide with Reaction Buffer
207. Dispense Coverslip
208. Rinse slide with Reaction Buffer
209. Fix volume on the slide with Reaction Buffer
210. Dispense Coverslip
211. Incubate per 4 minutes
212. Heat slide [36 °C] from Very Low Temperatures ( TS 3rd Antibody )
213. Dispense one aliquot of [Anti-Mouse NP] (Detection #8) and incubate per [0 ore 8 min]
214. Rinse slide with Reaction Buffer
215. Fix volume on the slide with Reaction Buffer
216. Dispense Coverslip
217. Disable slides heating
218. Heat slides a 37°C
219. Rinse slide with Reaction Buffer
220. Fix volume on the slide with Reaction Buffer
221. Dispense Coverslip
222. Rinse slide with Reaction Buffer
223. Fix volume on the slide with Reaction Buffer
224. Dispense Coverslip
225. [ Select Multimer ]

Tappa Operazione eseguita

1. Dispense one [Anti-NP AP] aliquot (Conjugate #3) and incubate per [8 minutes]
2. Rinse slide with Reaction Buffer
3. Fix volume on the slide with Reaction Buffer
4. Dispense Coverslip
5. Rinse slide with Reaction Buffer
6. Fix volume on the slide with Reaction Buffer
7. Dispense Coverslip
8. Rinse slide with Reaction Buffer
9. Fix volume on the slide with Reaction Buffer
10. Dispense Coverslip
11. Rinse slide with EZ Prep
12. Fix slide volume with EZ Prep
13. Dispense Coverslip
14. Rinse slide with EZ Prep
15. Fix slide volume with EZ Prep
16. Dispense Coverslip
17. Dispense one Activator CM aliquot, and incubate per 4 minutes
18. Dispense one aliquot of NBT CM and one of BCIP CM, and incubate 4 minutes
19. Incubat [0 ore 16 min] (Substrato)
20. Rinse slide with Reaction Buffer
21. Fix volume on the slide with Reaction Buffer
22. Dispense Coverslip
23. Rinse slide with Reaction Buffer
24. Fix volume on the slide with Reaction Buffer
25. Dispense Coverslip
26. Rinse slide with Reaction Buffer
27. Fix volume on the slide with Reaction Buffer
28. Dispense Coverslip
29. Dispense one aliquot [HEMATOXYLIN] (Counterstain) and incubate per [4 minutes]
30. Rinse slide with Reaction Buffer
31. Fix volume on the slide with Reaction Buffer
32. Dispense Coverslip

Step Operation

1. [ version 19 ]
2. [ For Research Use Only. Not intended for diagnostic purposes. ]
3. Enable mixer
4. Heat slides at 37°C
5. [ Delay refers to a time delayed start: Select time until run start ]
6. Disable mixer
7. [ 72°C is standard temperature]
8. Heat slide at [72°C] from Medium Temperature ( Dewaxing )
9. Incubate 4 minutes.
10. Fix EZ Prep volume (Discovery)
11. Rinse slide with EZ Prep
12. Fix EZ Prep volume (Discovery)
13. Dispense Coverslip
14. Rinse slide with EZ Prep
15. Fix EZ Prep volume (Discovery)
16. Dispense Coverslip
17. Enable mixer
18. Heat slides at 37°C
19. Rinse slide with EZ Prep
20. Dispense Cell Conditioner
21. Dispense Coverslip per CC
22. Heat slides [100°C] and incubate 4 minutes (Cell Conditioner 1)
23. Incubate 4 minutes
24. Incubate 8 minutes
25. Dispense Cell Conditioner 1
26. Dispense CC Coverslip medium, without Bar Code Blowoff
27. Incubate 8 minutes
28. Incubate 8 minutes
29. Dispense Cell Conditioner 1
30. Dispense CC medium Coverslip, senza Bar Code Blowoff
31. Incubate 8 minutes
32. Incubate 8 minutes
33. Dispense Cell Conditioner 1
34. Dispense CC medium Coverslip, without Bar Code Blowoff
35. Incubate 8 minutes
36. Dispense Cell Conditioner 1
37. Dispense CC medium Coverslip, without Bar Code Blowoff
38. Dispense Cell Conditioner 1
39. Dispense CC medium Coverslip, without Bar Code Blowoff
40. Dispense Cell Conditioner 1
41. Dispense CC medium Coverslip, without Bar Code Blowoff
42. Disable slides heating
43. Dispense Cell Conditioner 1
44. Dispense medium CC Coverslip, without Bar Code Blowoff
45. Heat slides 37°C
46. Rinse slide with Reaction Buffer
47. Fix volum on the slide with Reaction Buffer
48. Dispense Coverslip
49. [ Select an Inhibitor ]
50. [ NOTE: Inhibitor CM comes packaged with Chromomap DAB; InhibitorD comes packaged with DABMap ]
51. [ DISCOVERY Inhibitor is a stand alone product for use with all other HRP substrates ]
52. [ Inhibitor CM will be applied ]
53. Rinse slide with Reaction Buffer
54. Fix volume on the slide with Reaction Buffer
55. Dispense Coverslip
56. Rinse slide with Reaction Buffer
57. Fix volume on the slide with Reaction Buffer
58. Dispense Coverslip
59. Dispense one Inhibitor CM aliquot, then incubate [8 minutes]
60. Rinse slide with Reaction Buffer
61. Fix volume on the slide with Reaction Buffer
62. Dispense Coverslip
63. Disable slide heating
64. Disable mixer
65. Wait For Button ( Antibody )
66. Enable mixer
67. Heat slides at 37°C
68. Incubate 4 minutes
69. Rinse slide with Reaction Buffer
70. Fix volume on the slide with Reaction Buffer
71. Dispense Coverslip
72. Incubate 4 minutes
73. Heat slide [60°C] from Medium Temperature ( Primary Antibody )
74. Hand Apply ( Primary Antibody pStat1 ABCAM), and Incubate for [32 minutes]
75. Rinse slide with Reaction Buffer
76. Fix volume on the slide with Reaction Buffer
77. Dispense Coverslip
78. [ Inhibitor Solution will not be applied after the primary ]
79. Disableil slides heating
80. Heat slides at 37°C
81. [ Requires DETECTION dispensers ]
82. [ These selections may be used for haptenated linking antibodies ]
83. Rinse slide with Reaction Buffer
84. Fix volume on the slide with Reaction Buffer
85. Dispense Coverslip
86. Rinse slide with Reaction Buffer
87. Fix volume on the slide with Reaction Buffer
88. Dispense Coverslip
89. Rinse slide with Reaction Buffer
90. Fix volume on the slide with Reaction Buffer
91. Dispense Coverslip
92. Incubate 4 minutes
93. Heat slide a [36 °C] from Very Low Temperature ( Secondary Antibody )
94. Dispense one [Anti-Mouse HQ] aliquot (Revelation 1), then incubate [0 ore 8 min]
95. Rinse slide with Reaction Buffer
96. Fix volume on the slide with Reaction Buffer
97. Dispense Coverslip
98. Disable slides heating
99. Heat i slides a 37°C
100. Rinse slide with Reaction Buffer
101. Fix volume on the slide with Reaction Buffer
102. Dispense Coverslip
103. Rinse slide with Reaction Buffer
104. Fix volume on the slide with Reaction Buffer
105. Dispense Coverslip
106. [ Select Multimer ]
107. Dispense one [Anti-HQ HRP] aliquot (Conjugate #1) then incubate [8 minutes]
108. Rinse slide with Reaction Buffer
109. Fix volume on the slide with Reaction Buffer
110. Dispense Coverslip
111. Rinse slide with Reaction Buffer
112. Fix volume on the slide with Reaction Buffer
113. Dispense Coverslip
114. Rinse slide with Reaction Buffer
115. Fix volume on the slide with Reaction Buffer
116. Dispense Coverslip
117. Dispense one H2O2 CM aliquot, then incubate 4 minutes
118. Dispense one DAB CM aliquot, then incubate 8 minutes
119. Rinse slide with Reaction Buffer
120. Fix volume on the slide with Reaction Buffer
121. Dispense one Copper CM aliquot, add Coverslip then incubate 4 minutes
122. Rinse slide with Reaction Buffer
123. Fix volume on the slide with Reaction Buffer
124. Dispense Coverslip
125. [ DISC Inhibitor will not be applied ]
126. Rinse slide with Reaction Buffer
127. Fix volume on the slide with Reaction Buffer
128. Dispense Coverslip
129. Disable slides heating
130. Disable mixer
131. Wait For Button ( Primary Antibody CD163 LEICA)
132. Enable mixer
133. Heat slides at 37°C
134. Rinse slide with Reaction Buffer
135. Fix volume on the slide with Reaction Buffer
136. Dispense Coverslip
137. Heat slide [37°C] from Very Low Temperature ( Primary Antibody - (II) )
138. Hand Apply ( Primary Antibody - (II) ), and Incubate for [36 minutes]
139. Rinse slide with Reaction Buffer
140. Fix volume on the slide with Reaction Buffer
141. Dispense Coverslip
142. Disable slides heating.
143. Heat slides at 37°C
144. [ Requires DETECTION dispensers ]
145. Rinse slide with Reaction Buffer
146. Fix volume on the slide with Reaction Buffer
147. Dispense Coverslip
148. Rinse slide with Reaction Buffer
149. Fix volume on the slide with Reaction Buffer
150. Dispense Coverslip
151. Rinse slide with Reaction Buffer
152. Fix volume on the slide with Reaction Buffer
153. Dispense Coverslip
154. Incubate 4 minutes
155. Heat slide [36 °C] from Very Low Temperature ( 2nd Antibody - (II) )
156. Dispense one [Anti-Mouse NP] aliquote (Rivelazione 4) then incubate [0 ore 8 min]
157. Rinse slide with Reaction Buffer
158. Fix volume on the slide with Reaction Buffer
159. Dispense Coverslip
160. Disaable slides heating
161. Heat slides a 37°C
162. Rinse slide with Reaction Buffer
163. Fix volume on the slide with Reaction Buffer
164. Dispense Coverslip
165. Rinse slide with Reaction Buffer
166. Fix volume on the slide with Reaction Buffer
167. Dispense Coverslip
168. [ Select Multimer ]
169. Dispense one [Anti-NP AP] aliquot (Conjugate #2) then incubate per [8 minutes]
170. Rinse slide with Reaction Buffer
171. Fix volume on the slide with Reaction Buffer
172. Dispense Coverslip
173. Rinse slide with Reaction Buffer
174. Fix volume on the slide with Reaction Buffer
175. Dispense Coverslip
176. Rinse slide with Reaction Buffer
177. Fix volume on the slide with Reaction Buffer
178. Dispense one aliquot of DISC Naphthol and one of DISC Fast Red, add Coverslip and incubate per [16 minutes
179. Rinse slide with Reaction Buffer
180. Fix volume on the slide with Reaction Buffer
181. Dispense Coverslip
182. [ DISC Inhibitor will not be applied ]
183. Rinse slide with Reaction Buffer
184. Fix volume on the slide with Reaction Buffer
185. Dispense Coverslip
186. Disable slides heating
187. Disable mixer
188. Wait For Button ( Primary Antibody - (II) )
189. Enable mixer
190. Heat slides at 37°C
191. Rinse slide with Reaction Buffer
192. Fix volume on the slide with Reaction Buffer
193. Dispense Coverslip
194. Heat slide [36 °C] from Very Low Temperatures ( TS Primary Antibody )
195. Hand Apply ( TS Primary Antibody CD123 THERMOFISHER), and Incubate for [56 minutes]
196. Rinse slide with Reaction Buffer
197. Fix volume on the slide with Reaction Buffer
198. Dispense Coverslip
199. Disable slides heating
200. Heat slides a 37°C
201. [ Requires DETECTION dispensers ]
202. Rinse slide with Reaction Buffer
203. Fix volume on the slide with Reaction Buffer
204. Dispense Coverslip
205. Rinse slide with Reaction Buffer
206. Fix volume on the slide with Reaction Buffer
207. Dispense Coverslip
208. Rinse slide with Reaction Buffer
209. Fix volume on the slide with Reaction Buffer
210. Dispense Coverslip
211. Incubate per 4 minutes
212. Heat slide [36 °C] from Very Low Temperatures ( TS 3rd Antibody )
213. Dispense one aliquot of [Anti-Mouse NP] (Detection #8) and incubate per [0 ore 8 min]
214. Rinse slide with Reaction Buffer
215. Fix volume on the slide with Reaction Buffer
216. Dispense Coverslip
217. Disable slides heating
218. Heat slides a 37°C
219. Rinse slide with Reaction Buffer
220. Fix volume on the slide with Reaction Buffer
221. Dispense Coverslip
222. Rinse slide with Reaction Buffer
223. Fix volume on the slide with Reaction Buffer
224. Dispense Coverslip
225. [ Select Multimer ]

Tappa Operazione eseguita

1. Dispense one [Anti-NP AP] aliquot (Conjugate #3) and incubate per [8 minutes]
2. Rinse slide with Reaction Buffer
3. Fix volume on the slide with Reaction Buffer
4. Dispense Coverslip
5. Rinse slide with Reaction Buffer
6. Fix volume on the slide with Reaction Buffer
7. Dispense Coverslip
8. Rinse slide with Reaction Buffer
9. Fix volume on the slide with Reaction Buffer
10. Dispense Coverslip
11. Rinse slide with EZ Prep
12. Fix slide volume with EZ Prep
13. Dispense Coverslip
14. Rinse slide with EZ Prep
15. Fix slide volume with EZ Prep
16. Dispense Coverslip
17. Dispense one Activator CM aliquot, and incubate per 4 minutes
18. Dispense one aliquot of NBT CM and one of BCIP CM, and incubate 4 minutes
19. Incubat [0 ore 16 min] (Substrato)
20. Rinse slide with Reaction Buffer
21. Fix volume on the slide with Reaction Buffer
22. Dispense Coverslip
23. Rinse slide with Reaction Buffer
24. Fix volume on the slide with Reaction Buffer
25. Dispense Coverslip
26. Rinse slide with Reaction Buffer
27. Fix volume on the slide with Reaction Buffer
28. Dispense Coverslip
29. Dispense one aliquot [HEMATOXYLIN] (Counterstain) and incubate per [4 minutes]
30. Rinse slide with Reaction Buffer
31. Fix volume on the slide with Reaction Buffer
32. Dispense Coverslip
33. [ version 19 ]
34. [ For Research Use Only. Not intended for diagnostic purposes. ]
35. Enable mixer
36. Heat slides at 37°C
37. [ Delay refers to a time delayed start: Select time until run start ]
38. Disable mixer
39. [ 72°C is standard temperature]
40. Heat slide at [72°C] from Medium Temperature ( Dewaxing )
41. Incubate 4 minutes.
42. Fix EZ Prep volume (Discovery)
43. Rinse slide with EZ Prep
44. Fix EZ Prep volume (Discovery)
45. Dispense Coverslip
46. Rinse slide with EZ Prep
47. Fix EZ Prep volume (Discovery)
48. Dispense Coverslip
49. Enable mixer
50. Heat slides at 37°C
51. Rinse slide with EZ Prep
52. Dispense Cell Conditioner long 1
53. Dispense Coverslip per CC long
54. Heat slides [100°C] and incubate 4 minutes (Cell Conditioner 1)
55. Incubate 4 minutes
56. Incubate 8 minutes
57. Dispense Cell Conditioner 1
58. Dispense CC Coverslip medium, without Bar Code Blowoff
59. Incubate 8 minutes
60. Incubate 8 minutes
61. Dispense Cell Conditioner 1
62. Dispense CC medium Coverslip, senza Bar Code Blowoff
63. Incubate 8 minutes
64. Incubate 8 minutes
65. Dispense Cell Conditioner 1
66. Dispense CC medium Coverslip, without Bar Code Blowoff
67. Incubate 8 minutes
68. Dispense Cell Conditioner 1
69. Dispense CC medium Coverslip, without Bar Code Blowoff
70. Dispense Cell Conditioner 1
71. Dispense CC medium Coverslip, without Bar Code Blowoff
72. Dispense Cell Conditioner 1
73. Dispense CC medium Coverslip, without Bar Code Blowoff
74. Disable slides heating
75. Dispense Cell Conditioner 1
76. Dispense medium CC Coverslip, without Bar Code Blowoff
77. Heat slides 37°C
78. Rinse slide with Reaction Buffer
79. Fix volum on the slide with Reaction Buffer
80. Dispense Coverslip
81. [ Select an Inhibitor ]
82. [ NOTE: Inhibitor CM comes packaged with Chromomap DAB; InhibitorD comes packaged with DABMap ]
83. [ DISCOVERY Inhibitor is a stand alone product for use with all other HRP substrates ]
84. [ Inhibitor CM will be applied ]
85. Rinse slide with Reaction Buffer
86. Fix volume on the slide with Reaction Buffer
87. Dispense Coverslip
88. Rinse slide with Reaction Buffer
89. Fix volume on the slide with Reaction Buffer
90. Dispense Coverslip
91. Dispense one Inhibitor CM aliquot, then incubate [8 minutes]
92. Rinse slide with Reaction Buffer
93. Fix volume on the slide with Reaction Buffer
94. Dispense Coverslip
95. Disable slide heating
96. Disable mixer
97. Wait For Button ( Antibody )
98. Enable mixer
99. Heat slides at 37°C
100. Incubate 4 minutes
101. Rinse slide with Reaction Buffer
102. Fix volume on the slide with Reaction Buffer
103. Dispense Coverslip
104. Incubate 4 minutes
105. Heat slide [60°C] from Medium Temperature ( Primary Antibody )
106. Hand Apply ( Primary Antibody C-MAF ABCAM ), and Incubate for [32 minutes]
107. Rinse slide with Reaction Buffer
108. Fix volume on the slide with Reaction Buffer
109. Dispense Coverslip
110. [ Inhibitor Solution will not be applied after the primary ]
111. Disableil slides heating
112. Heat slides at 37°C
113. [ Requires DETECTION dispensers ]
114. [ These selections may be used for haptenated linking antibodies ]
115. Rinse slide with Reaction Buffer
116. Fix volume on the slide with Reaction Buffer
117. Dispense Coverslip
118. Rinse slide with Reaction Buffer
119. Fix volume on the slide with Reaction Buffer
120. Dispense Coverslip
121. Rinse slide with Reaction Buffer
122. Fix volume on the slide with Reaction Buffer
123. Dispense Coverslip
124. Incubate 4 minutes
125. Heat slide a [36 °C] from Very Low Temperature ( Secondary Antibody )
126. Dispense one [Anti-Rabbit HQ] aliquot (Revelation 1), then incubate [0 ore 8 min]
127. Rinse slide with Reaction Buffer
128. Fix volume on the slide with Reaction Buffer
129. Dispense Coverslip
130. Disable slides heating
131. Heat i slides a 37°C
132. Rinse slide with Reaction Buffer
133. Fix volume on the slide with Reaction Buffer
134. Dispense Coverslip
135. Rinse slide with Reaction Buffer
136. Fix volume on the slide with Reaction Buffer
137. Dispense Coverslip
138. [ Select Multimer ]
139. Dispense one [Anti-HQ HRP] aliquot (Conjugate #1) then incubate [8 minutes]
140. Rinse slide with Reaction Buffer
141. Fix volume on the slide with Reaction Buffer
142. Dispense Coverslip
143. Rinse slide with Reaction Buffer
144. Fix volume on the slide with Reaction Buffer
145. Dispense Coverslip
146. Rinse slide with Reaction Buffer
147. Fix volume on the slide with Reaction Buffer
148. Dispense Coverslip
149. Dispense one H2O2 CM aliquot, then incubate 4 minutes
150. Dispense one DAB CM aliquot, then incubate 8 minutes
151. Rinse slide with Reaction Buffer
152. Fix volume on the slide with Reaction Buffer
153. Dispense one Copper CM aliquot, add Coverslip then incubate 4 minutes
154. Rinse slide with Reaction Buffer
155. Fix volume on the slide with Reaction Buffer
156. Dispense Coverslip
157. [ DISC Inhibitor will not be applied ]
158. Rinse slide with Reaction Buffer
159. Fix volume on the slide with Reaction Buffer
160. Dispense Coverslip
161. Disable slides heating
162. Disable mixer
163. Wait For Button ( Primary Antibody - (II) )
164. Enable mixer
165. Heat slides at 37°C
166. Rinse slide with Reaction Buffer
167. Fix volume on the slide with Reaction Buffer
168. Dispense Coverslip
169. Heat slide [37°C] from Very Low Temperature ( Primary Antibody - (II) )
170. Hand Apply ( Primary Antibody - (II) CD163 ABCAM), and Incubate for [36 minutes]
171. Rinse slide with Reaction Buffer
172. Fix volume on the slide with Reaction Buffer
173. Dispense Coverslip
174. Disable slides heating.
175. Heat slides at 37°C
176. [ Requires DETECTION dispensers ]
177. Rinse slide with Reaction Buffer
178. Fix volume on the slide with Reaction Buffer
179. Dispense Coverslip
180. Rinse slide with Reaction Buffer
181. Fix volume on the slide with Reaction Buffer
182. Dispense Coverslip
183. Rinse slide with Reaction Buffer
184. Fix volume on the slide with Reaction Buffer
185. Dispense Coverslip
186. Incubate 4 minutes
187. Heat slide [36 °C] from Very Low Temperature ( 2nd Antibody - (II) )
188. Dispense one [Anti-Mouse NP] aliquote (Rivelazione 4) then incubate [0 ore 8 min]
189. Rinse slide with Reaction Buffer
190. Fix volume on the slide with Reaction Buffer
191. Dispense Coverslip
192. Disaable slides heating
193. Heat slides a 37°C
194. Rinse slide with Reaction Buffer
195. Fix volume on the slide with Reaction Buffer
196. Dispense Coverslip
197. Rinse slide with Reaction Buffer
198. Fix volume on the slide with Reaction Buffer
199. Dispense Coverslip
200. [ Select Multimer ]
201. Dispense one [Anti-NP AP] aliquot (Conjugate #2) then incubate per [8 minutes]
202. Rinse slide with Reaction Buffer
203. Fix volume on the slide with Reaction Buffer
204. Dispense Coverslip
205. Rinse slide with Reaction Buffer
206. Fix volume on the slide with Reaction Buffer
207. Dispense Coverslip
208. Rinse slide with Reaction Buffer
209. Fix volume on the slide with Reaction Buffer
210. Dispense one aliquot of DISC Naphthol and one of DISC Fast Red, add Coverslip and incubate per [16 minutes
211. Rinse slide with Reaction Buffer
212. Fix volume on the slide with Reaction Buffer
213. Dispense Coverslip
214. [ DISC Inhibitor will not be applied ]
215. Rinse slide with Reaction Buffer
216. Fix volume on the slide with Reaction Buffer
217. Dispense Coverslip
218. Disable slides heating
219. Disable mixer
220. Wait For Button ( Primary Antibody - (II) )
221. Enable mixer
222. Heat slides at 37°C
223. Rinse slide with Reaction Buffer
224. Fix volume on the slide with Reaction Buffer
225. Dispense Coverslip
226. Heat slide [36 °C] from Very Low Temperatures ( TS Primary Antibody )
227. Hand Apply ( TS Primary Antibody CD68KP1 VENTANA ROCHE), and Incubate for [56 minutes]
228. Rinse slide with Reaction Buffer
229. Fix volume on the slide with Reaction Buffer
230. Dispense Coverslip
231. Disable slides heating
232. Heat slides a 37°C
233. [ Requires DETECTION dispensers ]
234. Rinse slide with Reaction Buffer
235. Fix volume on the slide with Reaction Buffer
236. Dispense Coverslip
237. Rinse slide with Reaction Buffer
238. Fix volume on the slide with Reaction Buffer
239. Dispense Coverslip
240. Rinse slide with Reaction Buffer
241. Fix volume on the slide with Reaction Buffer
242. Dispense Coverslip
243. Incubate per 4 minutes
244. Heat slide [36 °C] from Very Low Temperatures ( TS 3rd Antibody )
245. Dispense one aliquot of [Anti-Mouse NP] (Detection #8) and incubate per [0 ore 8 min]
246. Rinse slide with Reaction Buffer
247. Fix volume on the slide with Reaction Buffer
248. Dispense Coverslip
249. Disable slides heating
250. Heat slides a 37°C
251. Rinse slide with Reaction Buffer
252. Fix volume on the slide with Reaction Buffer
253. Dispense Coverslip
254. Rinse slide with Reaction Buffer
255. Fix volume on the slide with Reaction Buffer
256. Dispense Coverslip
257. [ Select Multimer ]

**Tappa Operazione eseguita**

1. Dispense one [Anti-NP AP] aliquot (Conjugate #3) and incubate per [8 minutes]
2. Rinse slide with Reaction Buffer
3. Fix volume on the slide with Reaction Buffer
4. Dispense Coverslip
5. Rinse slide with Reaction Buffer
6. Fix volume on the slide with Reaction Buffer
7. Dispense Coverslip
8. Rinse slide with Reaction Buffer
9. Fix volume on the slide with Reaction Buffer
10. Dispense Coverslip
11. Rinse slide with EZ Prep
12. Fix slide volume with EZ Prep
13. Dispense Coverslip
14. Rinse slide with EZ Prep
15. Fix slide volume with EZ Prep
16. Dispense Coverslip
17. Dispense one Activator CM aliquot, and incubate per 4 minutes
18. Dispense one aliquot of NBT CM and one of BCIP CM, and incubate 4 minutes
19. Incubat [0 ore 16 min] (Substrato)
20. Rinse slide with Reaction Buffer
21. Fix volume on the slide with Reaction Buffer
22. Dispense Coverslip
23. Rinse slide with Reaction Buffer
24. Fix volume on the slide with Reaction Buffer
25. Dispense Coverslip
26. Rinse slide with Reaction Buffer
27. Fix volume on the slide with Reaction Buffer
28. Dispense Coverslip
29. Dispense one aliquot [HEMATOXYLIN] (Counterstain) and incubate per [4 minutes]
30. Rinse slide with Reaction Buffer
31. Fix volume on the slide with Reaction Buffer
32. Dispense Coverslip

Step Operation

1. [ version 19 ]
2. [ For Research Use Only. Not intended for diagnostic purposes. ]
3. Enable mixer
4. Heat slides at 37°C
5. [ Delay refers to a time delayed start: Select time until run start ]
6. Disable mixer
7. [ 72°C is standard temperature]
8. Heat slide at [72°C] from Medium Temperature ( Dewaxing )
9. Incubate 4 minutes.
10. Fix EZ Prep volume (Discovery)
11. Rinse slide with EZ Prep
12. Fix EZ Prep volume (Discovery)
13. Dispense Coverslip
14. Rinse slide with EZ Prep
15. Fix EZ Prep volume (Discovery)
16. Dispense Coverslip
17. Enable mixer
18. Heat slides at 37°C
19. Rinse slide with EZ Prep
20. Dispense Cell Conditioner
21. Dispense Coverslip per CC
22. Heat slides [100°C] and incubate 4 minutes (Cell Conditioner 1)
23. Incubate 4 minutes
24. Incubate 8 minutes
25. Dispense Cell Conditioner 1
26. Dispense CC Coverslip medium, without Bar Code Blowoff
27. Incubate 8 minutes
28. Incubate 8 minutes
29. Dispense Cell Conditioner 1
30. Dispense CC medium Coverslip, senza Bar Code Blowoff
31. Incubate 8 minutes
32. Incubate 8 minutes
33. Dispense Cell Conditioner 1
34. Dispense CC medium Coverslip, without Bar Code Blowoff
35. Incubate 8 minutes
36. Dispense Cell Conditioner 1
37. Dispense CC medium Coverslip, without Bar Code Blowoff
38. Dispense Cell Conditioner 1
39. Dispense CC medium Coverslip, without Bar Code Blowoff
40. Dispense Cell Conditioner 1
41. Dispense CC medium Coverslip, without Bar Code Blowoff
42. Disable slides heating
43. Dispense Cell Conditioner 1
44. Dispense medium CC Coverslip, without Bar Code Blowoff
45. Heat slides 37°C
46. Rinse slide with Reaction Buffer
47. Fix volum on the slide with Reaction Buffer
48. Dispense Coverslip
49. [ Select an Inhibitor ]
50. [ NOTE: Inhibitor CM comes packaged with Chromomap DAB; InhibitorD comes packaged with DABMap ]
51. [ DISCOVERY Inhibitor is a stand alone product for use with all other HRP substrates ]
52. [ Inhibitor CM will be applied ]
53. Rinse slide with Reaction Buffer
54. Fix volume on the slide with Reaction Buffer
55. Dispense Coverslip
56. Rinse slide with Reaction Buffer
57. Fix volume on the slide with Reaction Buffer
58. Dispense Coverslip
59. Dispense one Inhibitor CM aliquot, then incubate [8 minutes]
60. Rinse slide with Reaction Buffer
61. Fix volume on the slide with Reaction Buffer
62. Dispense Coverslip
63. Disable slide heating
64. Disable mixer
65. Wait For Button ( Antibody )
66. Enable mixer
67. Heat slides at 37°C
68. Incubate 4 minutes
69. Rinse slide with Reaction Buffer
70. Fix volume on the slide with Reaction Buffer
71. Dispense Coverslip
72. Incubate 4 minutes
73. Heat slide [60°C] from Medium Temperature ( Primary Antibody )
74. Hand Apply ( Primary Antibody CD25 DAKO), and Incubate for [32 minutes]
75. Rinse slide with Reaction Buffer
76. Fix volume on the slide with Reaction Buffer
77. Dispense Coverslip
78. [ Inhibitor Solution will not be applied after the primary ]
79. Disableil slides heating
80. Heat slides at 37°C
81. [ Requires DETECTION dispensers ]
82. [ These selections may be used for haptenated linking antibodies ]
83. Rinse slide with Reaction Buffer
84. Fix volume on the slide with Reaction Buffer
85. Dispense Coverslip
86. Rinse slide with Reaction Buffer
87. Fix volume on the slide with Reaction Buffer
88. Dispense Coverslip
89. Rinse slide with Reaction Buffer
90. Fix volume on the slide with Reaction Buffer
91. Dispense Coverslip
92. Incubate 4 minutes
93. Heat slide a [36 °C] from Very Low Temperature ( Secondary Antibody )
94. Dispense one [Anti-Mouse HQ] aliquot (Revelation 1), then incubate [0 ore 8 min]
95. Rinse slide with Reaction Buffer
96. Fix volume on the slide with Reaction Buffer
97. Dispense Coverslip
98. Disable slides heating
99. Heat i slides a 37°C
100. Rinse slide with Reaction Buffer
101. Fix volume on the slide with Reaction Buffer
102. Dispense Coverslip
103. Rinse slide with Reaction Buffer
104. Fix volume on the slide with Reaction Buffer
105. Dispense Coverslip
106. [ Select Multimer ]
107. Dispense one [Anti-HQ HRP] aliquot (Conjugate #1) then incubate [8 minutes]
108. Rinse slide with Reaction Buffer
109. Fix volume on the slide with Reaction Buffer
110. Dispense Coverslip
111. Rinse slide with Reaction Buffer
112. Fix volume on the slide with Reaction Buffer
113. Dispense Coverslip
114. Rinse slide with Reaction Buffer
115. Fix volume on the slide with Reaction Buffer
116. Dispense Coverslip
117. Dispense one H2O2 CM aliquot, then incubate 4 minutes
118. Dispense one DAB CM aliquot, then incubate 8 minutes
119. Rinse slide with Reaction Buffer
120. Fix volume on the slide with Reaction Buffer
121. Dispense one Copper CM aliquot, add Coverslip then incubate 4 minutes
122. Rinse slide with Reaction Buffer
123. Fix volume on the slide with Reaction Buffer
124. Dispense Coverslip
125. [ DISC Inhibitor will not be applied ]
126. Rinse slide with Reaction Buffer
127. Fix volume on the slide with Reaction Buffer
128. Dispense Coverslip
129. Disable slides heating
130. Disable mixer
131. Wait For Button ( Primary Antibody CD4 VENTANA)
132. Enable mixer
133. Heat slides at 37°C
134. Rinse slide with Reaction Buffer
135. Fix volume on the slide with Reaction Buffer
136. Dispense Coverslip
137. Heat slide [37°C] from Very Low Temperature ( Primary Antibody - (II) )
138. Hand Apply ( Primary Antibody - (II) ), and Incubate for [36 minutes]
139. Rinse slide with Reaction Buffer
140. Fix volume on the slide with Reaction Buffer
141. Dispense Coverslip
142. Disable slides heating.
143. Heat slides at 37°C
144. [ Requires DETECTION dispensers ]
145. Rinse slide with Reaction Buffer
146. Fix volume on the slide with Reaction Buffer
147. Dispense Coverslip
148. Rinse slide with Reaction Buffer
149. Fix volume on the slide with Reaction Buffer
150. Dispense Coverslip
151. Rinse slide with Reaction Buffer
152. Fix volume on the slide with Reaction Buffer
153. Dispense Coverslip
154. Incubate 4 minutes
155. Heat slide [36 °C] from Very Low Temperature ( 2nd Antibody - (II) )
156. Dispense one [Anti-Mouse NP] aliquote (Rivelazione 4) then incubate [0 ore 8 min]
157. Rinse slide with Reaction Buffer
158. Fix volume on the slide with Reaction Buffer
159. Dispense Coverslip
160. Disaable slides heating
161. Heat slides a 37°C
162. Rinse slide with Reaction Buffer
163. Fix volume on the slide with Reaction Buffer
164. Dispense Coverslip
165. Rinse slide with Reaction Buffer
166. Fix volume on the slide with Reaction Buffer
167. Dispense Coverslip
168. [ Select Multimer ]
169. Dispense one [Anti-NP AP] aliquot (Conjugate #2) then incubate per [8 minutes]
170. Rinse slide with Reaction Buffer
171. Fix volume on the slide with Reaction Buffer
172. Dispense Coverslip
173. Rinse slide with Reaction Buffer
174. Fix volume on the slide with Reaction Buffer
175. Dispense Coverslip
176. Rinse slide with Reaction Buffer
177. Fix volume on the slide with Reaction Buffer
178. Dispense one aliquot of DISC Naphthol and one of DISC Fast Red, add Coverslip and incubate per [16 minutes
179. Rinse slide with Reaction Buffer
180. Fix volume on the slide with Reaction Buffer
181. Dispense Coverslip
182. [ DISC Inhibitor will not be applied ]
183. Rinse slide with Reaction Buffer
184. Fix volume on the slide with Reaction Buffer
185. Dispense Coverslip
186. Disable slides heating
187. Disable mixer
188. Wait For Button ( Primary Antibody - (II) )
189. Enable mixer
190. Heat slides at 37°C
191. Rinse slide with Reaction Buffer
192. Fix volume on the slide with Reaction Buffer
193. Dispense Coverslip
194. Heat slide [36 °C] from Very Low Temperatures ( TS Primary Antibody )
195. Hand Apply ( TS Primary Antibody FOXP3 SANTA CRUZ), and Incubate for [56 minutes]
196. Rinse slide with Reaction Buffer
197. Fix volume on the slide with Reaction Buffer
198. Dispense Coverslip
199. Disable slides heating
200. Heat slides a 37°C
201. [ Requires DETECTION dispensers ]
202. Rinse slide with Reaction Buffer
203. Fix volume on the slide with Reaction Buffer
204. Dispense Coverslip
205. Rinse slide with Reaction Buffer
206. Fix volume on the slide with Reaction Buffer
207. Dispense Coverslip
208. Rinse slide with Reaction Buffer
209. Fix volume on the slide with Reaction Buffer
210. Dispense Coverslip
211. Incubate per 4 minutes
212. Heat slide [36 °C] from Very Low Temperatures ( TS 3rd Antibody )
213. Dispense one aliquot of [Anti-Mouse NP] (Detection #8) and incubate per [0 ore 8 min]
214. Rinse slide with Reaction Buffer
215. Fix volume on the slide with Reaction Buffer
216. Dispense Coverslip
217. Disable slides heating
218. Heat slides a 37°C
219. Rinse slide with Reaction Buffer
220. Fix volume on the slide with Reaction Buffer
221. Dispense Coverslip
222. Rinse slide with Reaction Buffer
223. Fix volume on the slide with Reaction Buffer
224. Dispense Coverslip
225. [ Select Multimer ]

Tappa Operazione eseguita

1. Dispense one [Anti-NP AP] aliquot (Conjugate #3) and incubate per [8 minutes]
2. Rinse slide with Reaction Buffer
3. Fix volume on the slide with Reaction Buffer
4. Dispense Coverslip
5. Rinse slide with Reaction Buffer
6. Fix volume on the slide with Reaction Buffer
7. Dispense Coverslip
8. Rinse slide with Reaction Buffer
9. Fix volume on the slide with Reaction Buffer
10. Dispense Coverslip
11. Rinse slide with EZ Prep
12. Fix slide volume with EZ Prep
13. Dispense Coverslip
14. Rinse slide with EZ Prep
15. Fix slide volume with EZ Prep
16. Dispense Coverslip
17. Dispense one Activator CM aliquot, and incubate per 4 minutes
18. Dispense one aliquot of NBT CM and one of BCIP CM, and incubate 4 minutes
19. Incubat [0 ore 16 min] (Substrato)
20. Rinse slide with Reaction Buffer
21. Fix volume on the slide with Reaction Buffer
22. Dispense Coverslip
23. Rinse slide with Reaction Buffer
24. Fix volume on the slide with Reaction Buffer
25. Dispense Coverslip
26. Rinse slide with Reaction Buffer
27. Fix volume on the slide with Reaction Buffer
28. Dispense Coverslip
29. Dispense one aliquot [HEMATOXYLIN] (Counterstain) and incubate per [4 minutes]
30. Rinse slide with Reaction Buffer
31. Fix volume on the slide with Reaction Buffer
32. Dispense Coverslip

Step Operation

1. [ version 19 ]
2. [ For Research Use Only. Not intended for diagnostic purposes. ]
3. Enable mixer
4. Heat slides at 37°C
5. [ Delay refers to a time delayed start: Select time until run start ]
6. Disable mixer
7. [ 72°C is standard temperature]
8. Heat slide at [72°C] from Medium Temperature ( Dewaxing )
9. Incubate 4 minutes.
10. Fix EZ Prep volume (Discovery)
11. Rinse slide with EZ Prep
12. Fix EZ Prep volume (Discovery)
13. Dispense Coverslip
14. Rinse slide with EZ Prep
15. Fix EZ Prep volume (Discovery)
16. Dispense Coverslip
17. Enable mixer
18. Heat slides at 37°C
19. Rinse slide with EZ Prep
20. Dispense Cell Conditioner
21. Dispense Coverslip per CC
22. Heat slides [100°C] and incubate 4 minutes (Cell Conditioner 1)
23. Incubate 4 minutes
24. Incubate 8 minutes
25. Dispense Cell Conditioner 1
26. Dispense CC Coverslip medium, without Bar Code Blowoff
27. Incubate 8 minutes
28. Incubate 8 minutes
29. Dispense Cell Conditioner 1
30. Dispense CC medium Coverslip, senza Bar Code Blowoff
31. Incubate 8 minutes
32. Incubate 8 minutes
33. Dispense Cell Conditioner 1
34. Dispense CC medium Coverslip, without Bar Code Blowoff
35. Incubate 8 minutes
36. Dispense Cell Conditioner 1
37. Dispense CC medium Coverslip, without Bar Code Blowoff
38. Dispense Cell Conditioner 1
39. Dispense CC medium Coverslip, without Bar Code Blowoff
40. Dispense Cell Conditioner 1
41. Dispense CC medium Coverslip, without Bar Code Blowoff
42. Disable slides heating
43. Dispense Cell Conditioner 1
44. Dispense medium CC Coverslip, without Bar Code Blowoff
45. Heat slides 37°C

- One drop equals one reagent aliquot. Stampato 22/02/2021 10:20:06 Ventana Medical Systems, Inc., 1910 Innovation Park Drive Tucson, Arizona USA Pagina 1 di 6 VSS v12.4 Build 15110.1

1. Rinse slide with Reaction Buffer
2. Fix volum on the slide with Reaction Buffer
3. Dispense Coverslip
4. [ Select an Inhibitor ]
5. [ NOTE: Inhibitor CM comes packaged with Chromomap DAB; InhibitorD comes packaged with DABMap ]
6. [ DISCOVERY Inhibitor is a stand alone product for use with all other HRP substrates ]
7. [ Inhibitor CM will be applied ]
8. Rinse slide with Reaction Buffer
9. Fix volume on the slide with Reaction Buffer
10. Dispense Coverslip
11. Rinse slide with Reaction Buffer
12. Fix volume on the slide with Reaction Buffer
13. Dispense Coverslip
14. Dispense one Inhibitor CM aliquot, then incubate [8 minutes]
15. Rinse slide with Reaction Buffer
16. Fix volume on the slide with Reaction Buffer
17. Dispense Coverslip
18. Disable slide heating
19. Disable mixer
20. Wait For Button ( Antibody )
21. Enable mixer
22. Heat slides at 37°C
23. Incubate 4 minutes
24. Rinse slide with Reaction Buffer
25. Fix volume on the slide with Reaction Buffer
26. Dispense Coverslip
27. Incubate 4 minutes
28. Heat slide [60°C] from Medium Temperature ( Primary Antibody )
29. Hand Apply ( Primary Antibody PD 1 VENTANA), and Incubate for [32 minutes]
30. Rinse slide with Reaction Buffer
31. Fix volume on the slide with Reaction Buffer
32. Dispense Coverslip
33. [ Inhibitor Solution will not be applied after the primary ]
34. Disableil slides heating
35. Heat slides at 37°C
36. [ Requires DETECTION dispensers ]
37. [ These selections may be used for haptenated linking antibodies ]
38. Rinse slide with Reaction Buffer
39. Fix volume on the slide with Reaction Buffer
40. Dispense Coverslip
41. Rinse slide with Reaction Buffer
42. Fix volume on the slide with Reaction Buffer
43. Dispense Coverslip
44. Rinse slide with Reaction Buffer
45. Fix volume on the slide with Reaction Buffer

- One drop equals one reagent aliquot. Stampato 22/02/2021 10:20:06 Ventana Medical Systems, Inc., 1910 Innovation Park Drive Tucson, Arizona USA Pagina 2 di 6 VSS v12.4 Build 15110.1

1. Dispense Coverslip
2. Incubate 4 minutes
3. Heat slide a [36 °C] from Very Low Temperature ( Secondary Antibody )
4. Dispense one [Anti-Mouse HQ] aliquot (Revelation 1), then incubate [0 ore 8 min]
5. Rinse slide with Reaction Buffer
6. Fix volume on the slide with Reaction Buffer
7. Dispense Coverslip
8. Disable slides heating
9. Heat i slides a 37°C
10. Rinse slide with Reaction Buffer
11. Fix volume on the slide with Reaction Buffer
12. Dispense Coverslip
13. Rinse slide with Reaction Buffer
14. Fix volume on the slide with Reaction Buffer
15. Dispense Coverslip
16. [ Select Multimer ]
17. Dispense one [Anti-HQ HRP] aliquot (Conjugate #1) then incubate [8 minutes]
18. Rinse slide with Reaction Buffer
19. Fix volume on the slide with Reaction Buffer
20. Dispense Coverslip
21. Rinse slide with Reaction Buffer
22. Fix volume on the slide with Reaction Buffer
23. Dispense Coverslip
24. Rinse slide with Reaction Buffer
25. Fix volume on the slide with Reaction Buffer
26. Dispense Coverslip
27. Dispense one H2O2 CM aliquot, then incubate 4 minutes
28. Dispense one DAB CM aliquot, then incubate 8 minutes
29. Rinse slide with Reaction Buffer
30. Fix volume on the slide with Reaction Buffer
31. Dispense one Copper CM aliquot, add Coverslip then incubate 4 minutes
32. Rinse slide with Reaction Buffer
33. Fix volume on the slide with Reaction Buffer
34. Dispense Coverslip
35. [ DISC Inhibitor will not be applied ]
36. Rinse slide with Reaction Buffer
37. Fix volume on the slide with Reaction Buffer
38. Dispense Coverslip
39. Disable slides heating
40. Disable mixer
41. Wait For Button ( Primary Antibody CD8 LEICA)
42. Enable mixer
43. Heat slides at 37°C
44. Rinse slide with Reaction Buffer
45. Fix volume on the slide with Reaction Buffer
46. Dispense Coverslip
47. Heat slide [37°C] from Very Low Temperature ( Primary Antibody - (II) )
48. Hand Apply ( Primary Antibody - (II) ), and Incubate for [36 minutes]
49. Rinse slide with Reaction Buffer
50. Fix volume on the slide with Reaction Buffer
51. Dispense Coverslip
52. Disable slides heating.
53. Heat slides at 37°C
54. [ Requires DETECTION dispensers ]
55. Rinse slide with Reaction Buffer
56. Fix volume on the slide with Reaction Buffer
57. Dispense Coverslip
58. Rinse slide with Reaction Buffer
59. Fix volume on the slide with Reaction Buffer
60. Dispense Coverslip
61. Rinse slide with Reaction Buffer
62. Fix volume on the slide with Reaction Buffer
63. Dispense Coverslip
64. Incubate 4 minutes
65. Heat slide [36 °C] from Very Low Temperature ( 2nd Antibody - (II) )
66. Dispense one [Anti-Mouse NP] aliquote (Rivelazione 4) then incubate [0 ore 8 min]
67. Rinse slide with Reaction Buffer
68. Fix volume on the slide with Reaction Buffer
69. Dispense Coverslip
70. Disaable slides heating
71. Heat slides a 37°C
72. Rinse slide with Reaction Buffer
73. Fix volume on the slide with Reaction Buffer
74. Dispense Coverslip
75. Rinse slide with Reaction Buffer
76. Fix volume on the slide with Reaction Buffer
77. Dispense Coverslip
78. [ Select Multimer ]
79. Dispense one [Anti-NP AP] aliquot (Conjugate #2) then incubate per [8 minutes]
80. Rinse slide with Reaction Buffer
81. Fix volume on the slide with Reaction Buffer
82. Dispense Coverslip
83. Rinse slide with Reaction Buffer
84. Fix volume on the slide with Reaction Buffer
85. Dispense Coverslip
86. Rinse slide with Reaction Buffer
87. Fix volume on the slide with Reaction Buffer
88. Dispense one aliquot of DISC Naphthol and one of DISC Fast Red, add Coverslip and incubate per [16 minutes
89. Rinse slide with Reaction Buffer
90. Fix volume on the slide with Reaction Buffer
91. Dispense Coverslip
92. [ DISC Inhibitor will not be applied ]
93. Rinse slide with Reaction Buffer
94. Fix volume on the slide with Reaction Buffer
95. Dispense Coverslip
96. Disable slides heating
97. Disable mixer
98. Wait For Button ( Primary Antibody - (II) )
99. Enable mixer
100. Heat slides at 37°C
101. Rinse slide with Reaction Buffer
102. Fix volume on the slide with Reaction Buffer
103. Dispense Coverslip
104. Heat slide [36 °C] from Very Low Temperatures ( TS Primary Antibody )
105. Hand Apply ( TS Primary Antibody LAG3 ABCAM), and Incubate for [56 minutes]
106. Rinse slide with Reaction Buffer
107. Fix volume on the slide with Reaction Buffer
108. Dispense Coverslip
109. Disable slides heating
110. Heat slides a 37°C
111. [ Requires DETECTION dispensers ]
112. Rinse slide with Reaction Buffer
113. Fix volume on the slide with Reaction Buffer
114. Dispense Coverslip
115. Rinse slide with Reaction Buffer
116. Fix volume on the slide with Reaction Buffer
117. Dispense Coverslip
118. Rinse slide with Reaction Buffer
119. Fix volume on the slide with Reaction Buffer
120. Dispense Coverslip
121. Incubate per 4 minutes
122. Heat slide [36 °C] from Very Low Temperatures ( TS 3rd Antibody )
123. Dispense one aliquot of [Anti-Mouse NP] (Detection #8) and incubate per [0 ore 8 min]
124. Rinse slide with Reaction Buffer
125. Fix volume on the slide with Reaction Buffer
126. Dispense Coverslip
127. Disable slides heating
128. Heat slides a 37°C
129. Rinse slide with Reaction Buffer
130. Fix volume on the slide with Reaction Buffer
131. Dispense Coverslip
132. Rinse slide with Reaction Buffer
133. Fix volume on the slide with Reaction Buffer
134. Dispense Coverslip
135. [ Select Multimer ]

Tappa Operazione eseguita

1. Dispense one [Anti-NP AP] aliquot (Conjugate #3) and incubate per [8 minutes]
2. Rinse slide with Reaction Buffer
3. Fix volume on the slide with Reaction Buffer
4. Dispense Coverslip
5. Rinse slide with Reaction Buffer
6. Fix volume on the slide with Reaction Buffer
7. Dispense Coverslip
8. Rinse slide with Reaction Buffer
9. Fix volume on the slide with Reaction Buffer
10. Dispense Coverslip
11. Rinse slide with EZ Prep
12. Fix slide volume with EZ Prep
13. Dispense Coverslip
14. Rinse slide with EZ Prep
15. Fix slide volume with EZ Prep
16. Dispense Coverslip
17. Dispense one Activator CM aliquot, and incubate per 4 minutes
18. Dispense one aliquot of NBT CM and one of BCIP CM, and incubate 4 minutes
19. Incubat [0 ore 16 min] (Substrato)
20. Rinse slide with Reaction Buffer
21. Fix volume on the slide with Reaction Buffer
22. Dispense Coverslip
23. Rinse slide with Reaction Buffer
24. Fix volume on the slide with Reaction Buffer
25. Dispense Coverslip
26. Rinse slide with Reaction Buffer
27. Fix volume on the slide with Reaction Buffer
28. Dispense Coverslip
29. Dispense one aliquot [HEMATOXYLIN] (Counterstain) and incubate per [4 minutes]
30. Rinse slide with Reaction Buffer
31. Fix volume on the slide with Reaction Buffer
32. Dispense Coverslip
33. [ version 19 ]
34. [ For Research Use Only. Not intended for diagnostic purposes. ]
35. Mixer skills
36. Heat the slide at 37°C
37. [ Delay refers to a time delayed start: Select time until run start ]
38. Disability mixer
39. [ 72°C standard temperature ]
40. Heat the slide at [72°C] (dewaxing)
41. Incubate for 4 minutes
42. EZ Prep (Discovery)
43. Rinse the slide with EZ Prep
44. Dispense Coverslip
45. Rinse the slide with EZ Prep
46. Dispense Coverslip
47. Heat the slide at 37°C
48. Dispense Cell Conditioner lungo 1
49. Dispense Coverslip per CC1
50. Heat the slide at [100°C] e incubate for 4 minutes (Cell Conditioner 1)
51. Incubate for 4 minutes
52. Incubate for 8 minutes
53. Dispense Cell Conditioner 1
54. Dispense CC Coverslip, without Bar Code Blowoff
55. Incubate for 8 minuti
56. Dispense Cell Conditioner 1
57. Dispense CC Coverslip, without Bar Code Blowoff
58. Incubate for 8 minutes
59. Dispense Cell Conditioner 1
60. Dispense CC Coverslip, without Bar Code Blowoff
61. Incubate for 8 minutes
62. Dispense Cell Conditioner 1
63. Turn off the slide heating
64. Dispense Cell Conditioner 1
65. Dispense CC Coverslip, without Bar Code Blowoff
66. Heat the slide at 37°C
67. [ Select an Inhibitor ]
68. [ NOTE: Inhibitor CM comes packaged with Chromomap DAB; InhibitorD comes packaged with DABMap ]
69. [ DISCOVERY Inhibitor is a stand alone product for use with all other HRP substrates ]
70. [ Inhibitor CM will be applied ]
71. Rinse the slide with Reaction Buffer
72. Dispense an aliquot of Inhibitor CM, e incubate for [8 minuti]
73. Disability heating
74. Disability mixer
75. Wait For Button ( Anticorpo )
76. Mixer skills
77. Heat the slide at 37°C
78. Incubate for 4 minutes
79. Rinse the slide with Reaction Buffer
80. Incubate for 4 minutes
81. Heat the slide at [74°C] ( Antibody I )PDL-1 VENTANA SP263
82. Hand Apply (Antibody I), and Incubate for [36 minutes]
83. [ Inhibitor Solution will not be applied after the primary ]
84. Disabilty heating
85. Rinse the slide at 37°C
86. [ Requires DETECTION dispensers ]
87. [ These selections may be used for haptenated linking antibodies ]
88. Rinse the slide with Reaction Buffer
89. Incubate for 4 minutes
90. Rinse the slide at [36 °C] ( Anticorpo II)
91. [Anti-Mouse HQ] (DETECTION) and incubate for [0 ore 8 min]
92. [ Select Multimer ]
93. Dispense [Anti-HQ HRP] (Conjugate #1) for [8 minuti]
94. Rinse the slide with Reaction Buffer
95. Dispense H2O2 CM, and incubate for 4 minutes
96. Dispense DAB CM, and incubate for 8 minutes
97. Rinse the slide with Reaction Buffer
98. Dispense Copper CM, and incubate for 4 minutes
99. Rinse the slide with Reaction Buffer
100. [ DISC Inhibitor will not be applied ]
101. Risciacquare il vetrino con Reaction Buffer
102. Wait For Button ( Anticorpo primario - (II) )
103. Mixer skills
104. Rinse the slide at 37°C
105. Hand Apply ( Anticorpo primario - (II) CD163 MOUSE MONOCLONAL ABCAM ), and Incubate for [36 minuti]
106. Rinse the slide with Reaction Buffer
107. Heat up 37°C
108. [ Requires DETECTION dispensers ]
109. Rinse the slide with Reaction Buffer
110. Dispense [Anti-Rabbit HQ] and incubate for[0 ore 16 min]
111. Rinse the slide with Reaction Buffer
112. [ Select Multimer ]
113. Dispense [Anti-HQ HRP] (Conjugate #2) and incubate for [16 min]
114. Rinse the slide with Reaction Buffer
115. Dispense DISC Naphthol, DISC Fast Red, for [32 minutes
116. Rinse the slide with Reaction Buffer
117. Wait For Button ( Anticorpo primario - (II) )
118. Mixer skills
119. Heat the slide 37°C
120. Hand Apply ( TS Primary Antibody C-MAF ANTIBODY ab199424 abcam ), and Incubate for [36 minuti]
121. Rinse the slide with Reaction Buffer
122. Heat the slide 37°C
123. [ Requires DETECTION dispensers ]
124. Dispense [Anti-Rabbit HQ] (Detection #7) for [0 ore 16 min]
125. Rinse the slide with Reaction Buffer
126. [ Select Multimer ]
127. Dispense [Anti-HQ HRP] (Conjugate #3) for [16 min]
128. Rinse the slide with Reaction Buffer
129. Dispense Blue Substrate, for 0 ore 4 min
130. Dispense Blue H2O2, for [16 minuti]
131. Rinse the slide with Reaction Buffer
132. Dispense Blue Activator, for[16 min]
133. Rinse the slide with EZ Prep
134. Rinse the slide with Reaction Buffer
