# Supplementary figures and images for: Burkitt lymphoma with a granulomatous reaction: an M1/Th1‐polarised microenvironment is associated with controlled growth and spontaneous regression
Source: Histopathology. 2021 Jul 5;80(2):430–42. doi: 10.1111/his.14391 (PMC9291779; doi:10.1111/his.14391)

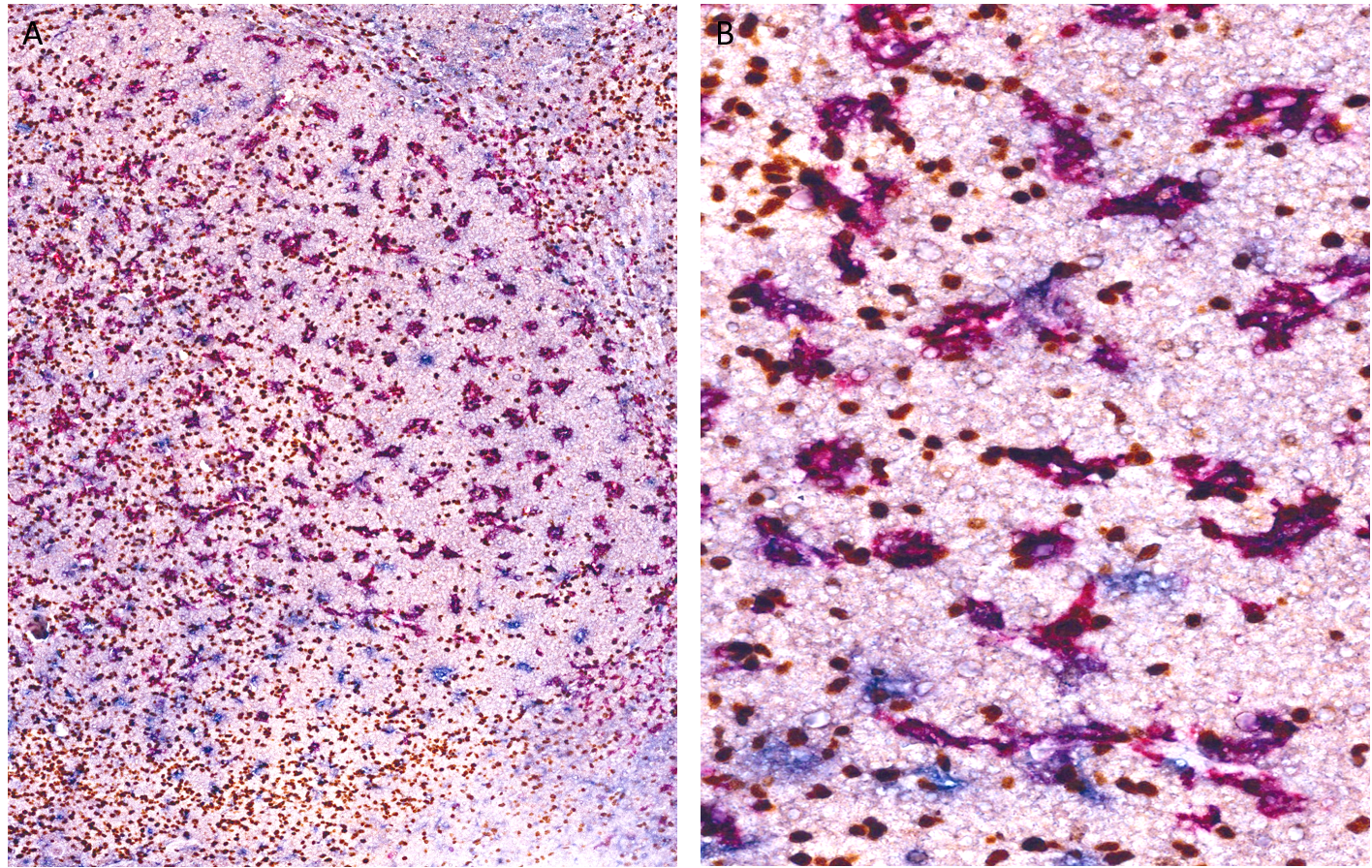

Supplement: Supplementary file 2 — Figure S1. High number of M2 macrophages in a reactive lymph node. [file HIS-80-430-s007.tif]

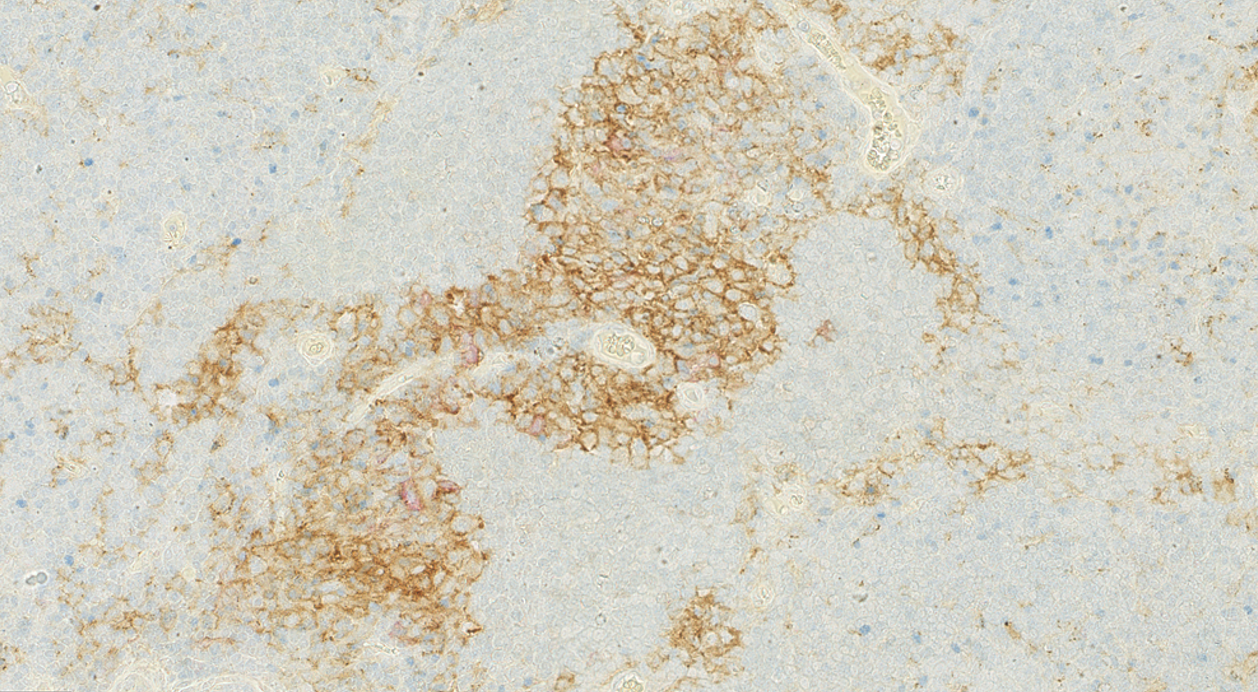

Supplement: Supplementary file 3 — Figure S2. Dichotomous role of IFN‐γ. [file HIS-80-430-s008.tif]

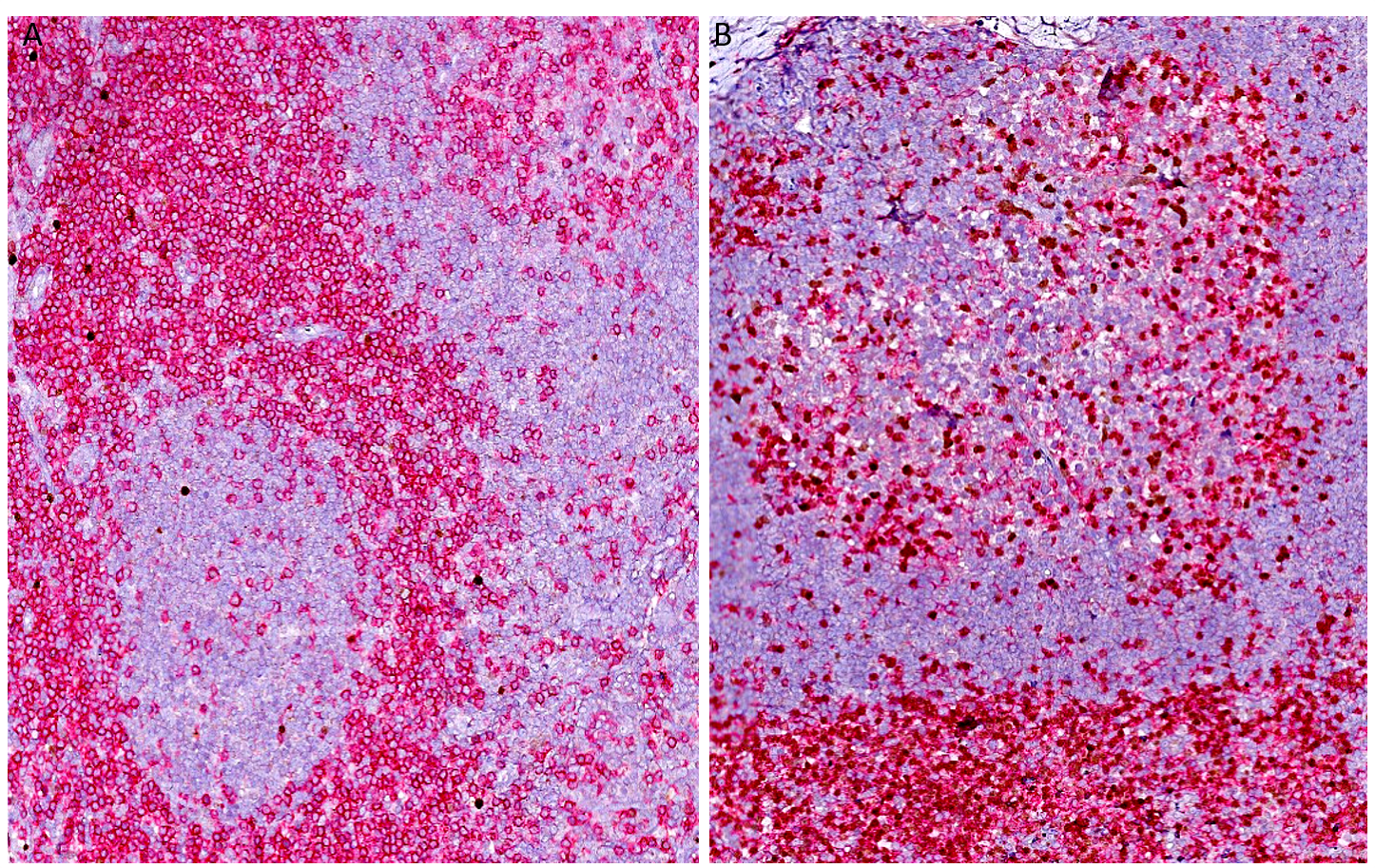

Supplement: Supplementary file 4 — Figure S3. Th1 and Th2 expression in the controls. [file HIS-80-430-s004.tif]

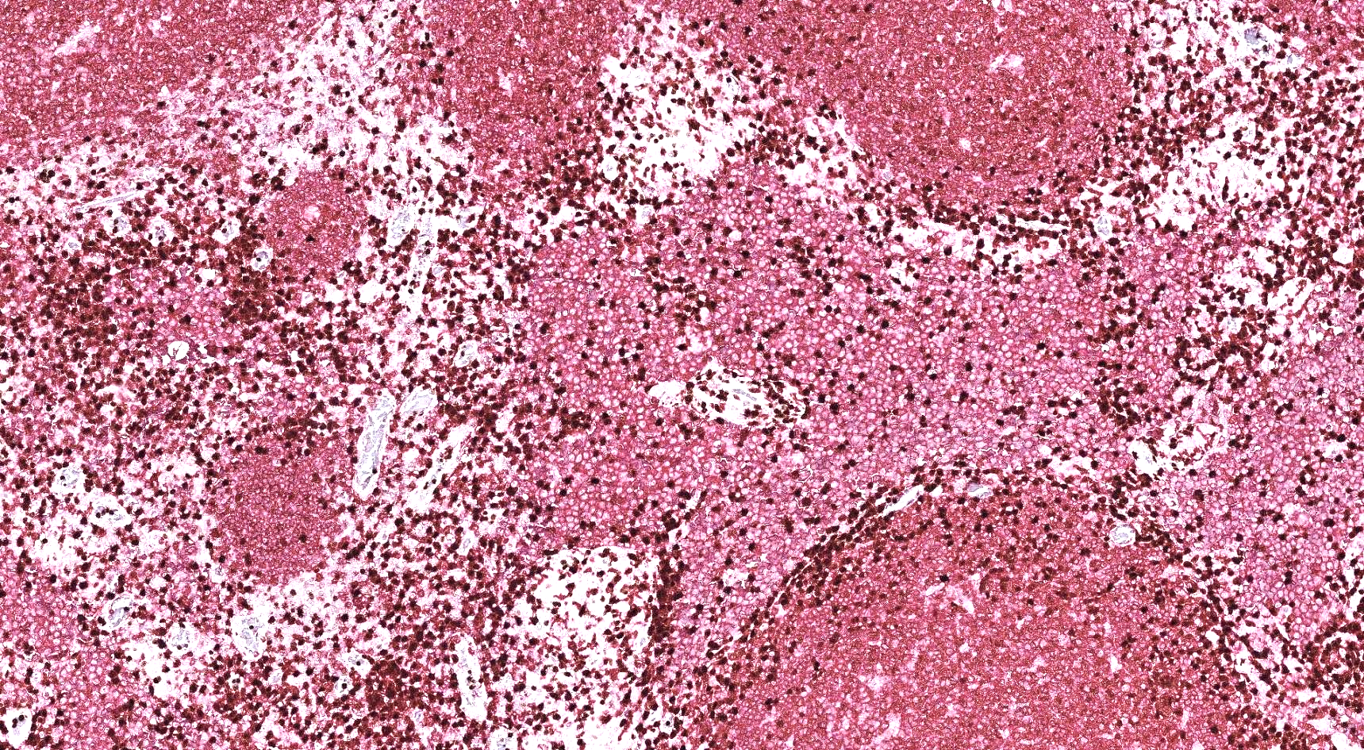

Supplement: Supplementary file 5 — Figure S4. Expression of T‐bet in B cells. [file HIS-80-430-s005.tif]

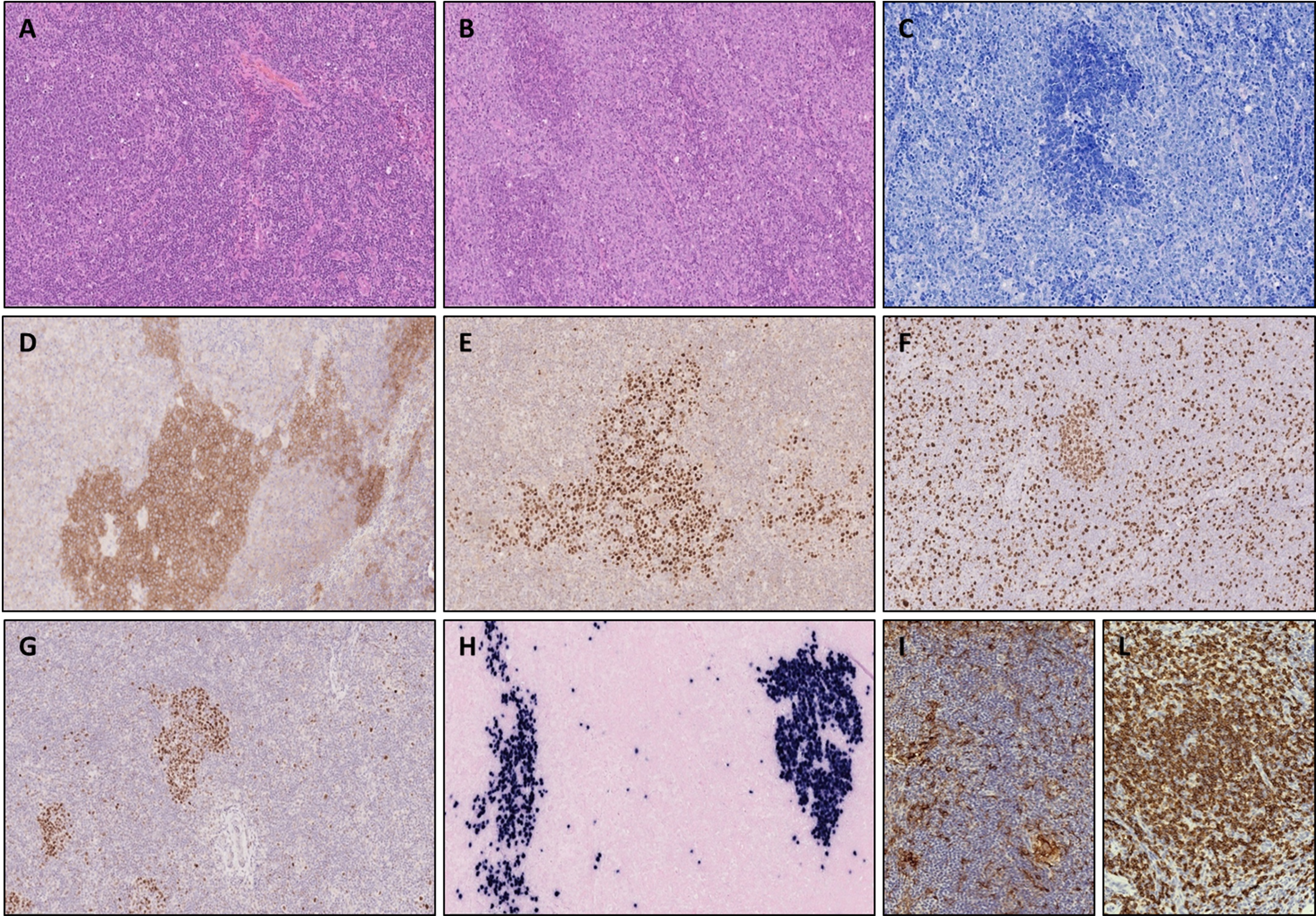

Supplement: Supplementary file 6 — Figure S5. Partial lymph node involvement by BL in an HIV‐positive patient. [file HIS-80-430-s003.tif]

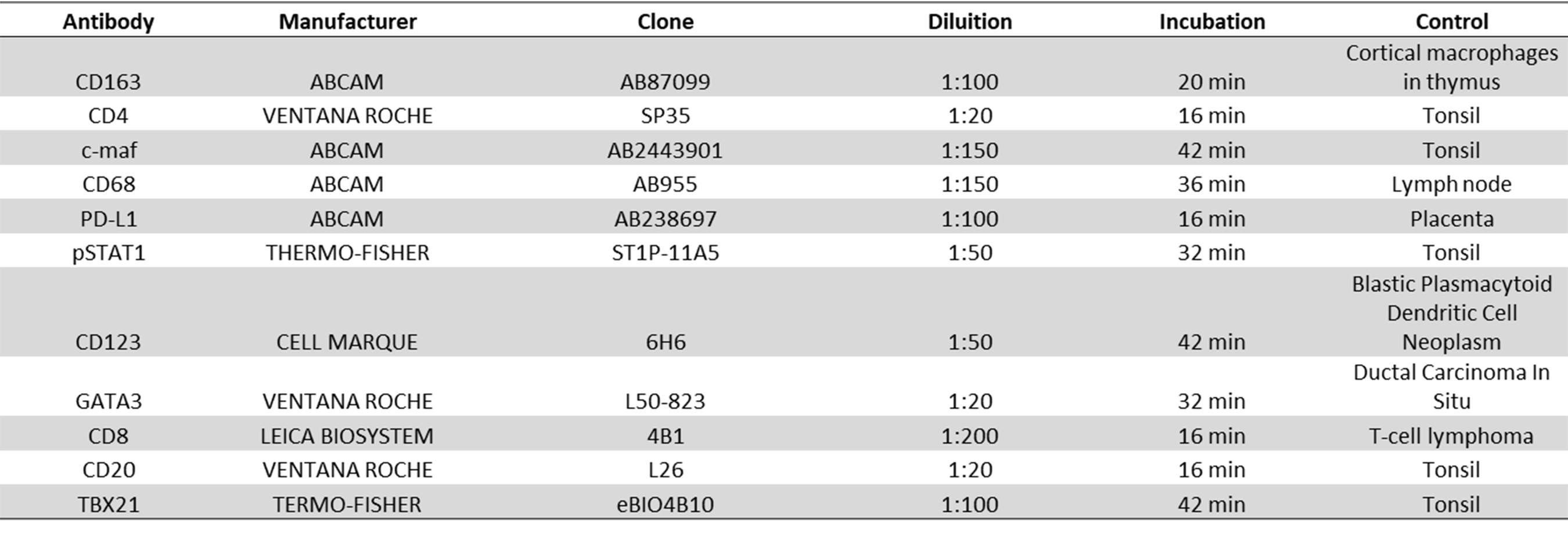

Supplement: Supplementary file 7 — Table S1. Antibody characteristics. [file HIS-80-430-s001.tif]

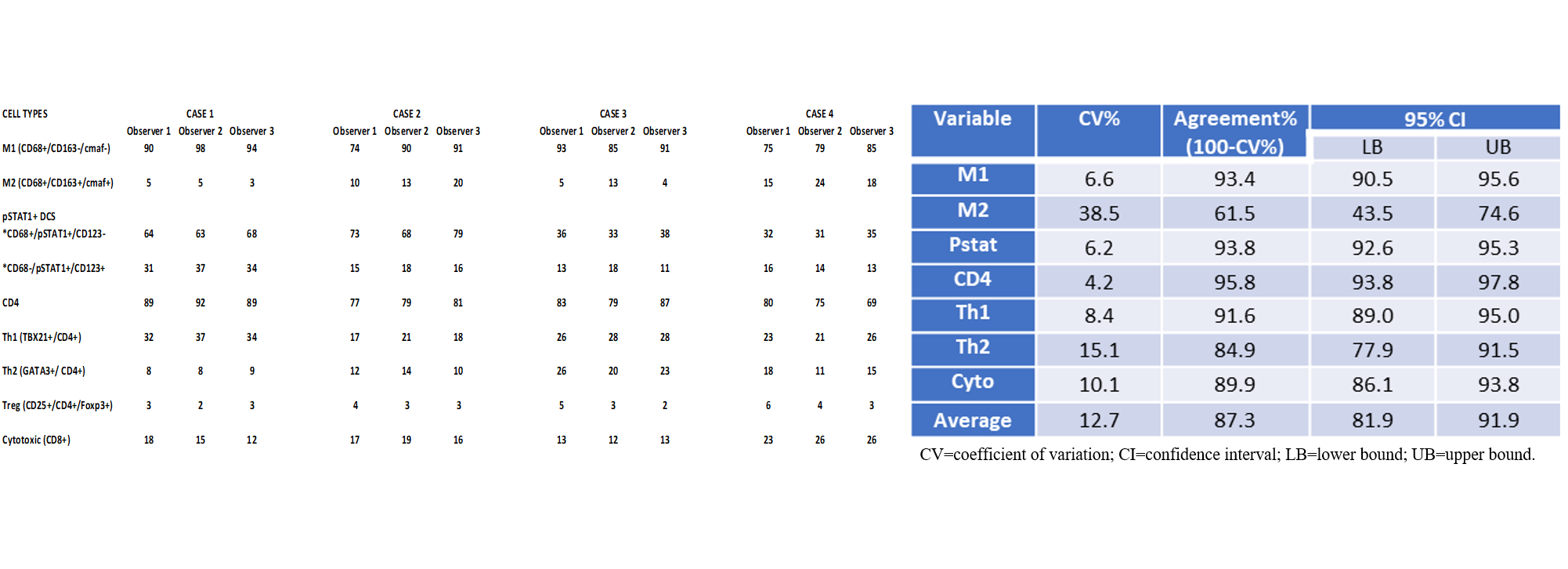

Supplement: Supplementary file 8 — Table S2. The individual scores of the observers and the agreement for all separate cell types. [file HIS-80-430-s006.tif]
